# The *Populus* holobiont: dissecting the effects of plant niches and genotype on the microbiome

**DOI:** 10.1186/s40168-018-0413-8

**Published:** 2018-02-12

**Authors:** M. A. Cregger, A. M. Veach, Z. K. Yang, M. J. Crouch, R. Vilgalys, G. A. Tuskan, C. W. Schadt

**Affiliations:** 1 Biosciences Division, Oak Ridge National Laboratory, 1 Bethel Valley Rd, Oak Ridge, TN 37831 USA; 2 Biology Department, Duke University, 130 Science Drive, Durham, NC 27708 USA; 3 Microbiology Department, University of Tennessee, M409 Walters Life Sciences, Knoxville, TN 37996 USA; 4 Present address: Department of Biochemistry & Molecular Biology, Brody School of Medicine, East Carolina Diabetes & Obesity Institute, East Carolina University, Greenville, NC USA

**Keywords:** *Populus deltoides*, *Populus trichocarpa × deltoides* hybrid, 16S rRNA, ITS2, Fungal pathogen

## Abstract

**Background:**

Microorganisms serve important functions within numerous eukaryotic host organisms. An understanding of the variation in the plant niche-level microbiome, from rhizosphere soils to plant canopies, is imperative to gain a better understanding of how both the structural and functional processes of microbiomes impact the health of the overall plant holobiome. Using *Populus* trees as a model ecosystem, we characterized the archaeal/bacterial and fungal microbiome across 30 different tissue-level niches within replicated *Populus deltoides* and hybrid *Populus trichocarpa × deltoides* individuals using 16S and ITS2 rRNA gene analyses.

**Results:**

Our analyses indicate that archaeal/bacterial and fungal microbiomes varied primarily across broader plant habitat classes (leaves, stems, roots, soils) regardless of plant genotype, except for fungal communities within leaf niches, which were greatly impacted by the host genotype. Differences between tree genotypes are evident in the elevated presence of two potential fungal pathogens, *Marssonina brunnea* and *Septoria* sp., on hybrid *P. trichocarpa × deltoides* trees which may in turn be contributing to divergence in overall microbiome composition. Archaeal/bacterial diversity increased from leaves, to stem, to root, and to soil habitats, whereas fungal diversity was the greatest in stems and soils.

**Conclusions:**

This study provides a holistic understanding of microbiome structure within a bioenergy relevant plant host, one of the most complete niche-level analyses of any plant. As such, it constitutes a detailed atlas or map for further hypothesis testing on the significance of individual microbial taxa within specific niches and habitats of *Populus* and a baseline for comparisons to other plant species.

**Electronic supplementary material:**

The online version of this article (10.1186/s40168-018-0413-8) contains supplementary material, which is available to authorized users.

## Background

Microorganisms are ubiquitous across all environments, yet we are just beginning to understand the role they play within ecosystems and in association with host organisms. Individual plant-associated microorganisms are known to aid in key functions across the entire plant, e.g., water and nutrient acquisition [1], stress response [2], suppression of pathogens [3], and reducing herbivory directly and through priming of host plant defenses [4]. As a result, the collective holobiomes or phytobiomes of plants are gaining increased attention [5, 6]. Although advances are being made in understanding microbiome composition within individual host habitats [7–13], little work has been conducted to holistically understand the variation in microbiome composition across the numerous potential microbial niches represented by multiple plant organ and tissue types [5].

*Populus* has become the model woody perennial organism for researchers interested in testing mechanistic hypotheses related to plant–microbe interactions. *Populus* is amenable to experimentation because of its fast growth rates and the ability to be propagated vegetatively. *Populus* has its full genome sequenced [14, 15]; therefore, the interaction between host genomic information and microbial associations is readily discernible. Further, understanding these interactions may be particularly important socioeconomically as poplar trees currently are cultivated for pulp and paper production [16, 17] and have potential as a cellulose-derived biofuel feedstock [14, 18–20].

Distinct microbiome composition of the *Populus* rhizosphere and root endosphere across environmental gradients [21, 22] and between *Populus* genotypes or species [23] has been demonstrated. Microbial community isolates from *Populus* have also been shown to enhance the health, growth, and development of their plant hosts [24–26]. Differentiation between root endosphere and rhizosphere microbial communities is likely due to selection of unique microbial consortia with the ability to penetrate and survive the host environment [21], although the strength of selection may differ between microbial groups. However, the degree of microbiome specificity across all plant-associated niches (i.e., soil to canopy) has not been effectively tested within *Populus* genotypes or between genotypes.

There are known pathogenic organisms that differentially attack *Populus* species and genotypes (e.g., *P. trichocarpa × deltoides*), and pathogen population abundance has been shown to vary among *Populus* species [27] and across genotypes within species [28]. Fungal pathogen abundance in *Populus* leaves has also been shown to be correlated with the co-occurrence of alternate fungal endophyte species that likely act as antagonists and competitors for both space and host resources [28, 29]. Understanding the basis of multi-pathogen resistance and the degree of pathogen interactions with the overall phytobiome may aid in the success of effectively growing *Populus* for pulp fiber and biofuel feedstock operations and understanding *Populus* contributions to ecosystem services.

Using *Populus* as a model system, this study seeks to understand how the collective communities of archaea/bacteria and fungi, or the microbiome, varied across habitats within a tree host from soil to tree canopy and between individual *Populus deltoides* and *Populus trichocarpa × deltoides* hybrids (ramets) under identical environmental conditions. We characterized microbial communities across 30 different plant-associated niches covering an extensive number of the aboveground and belowground tissue-level microbial habitats, as well as both shallow and deeper soil habitats (Additional file 1: Table S1), using amplicon 16S and ITS2 rRNA gene-targeted Illumina MiSeq sequencing. We hypothesized that due to differing microbial inoculum sources (i.e., air–leaf/stem interface vs. the root–soil interface) and environmental filtering mechanisms (e.g., tissue chemistry or exudates in roots [30]), microbiome niche-level composition for archaea/bacteria and fungi would vary across the landscape represented by the ecosystem of whole trees. Further, due to differences in susceptibility of different *Populus* species to fungal pathogen infection, we hypothesized that microbial communities would differ between *Populus deltoides* and the *Populus trichocarpa × deltoides* hybrid.

## Methods

### Study location and sampling methods

Trees used in this study were harvested from an experimental cultivar trial in Blount County Tennessee at a site managed by the University of Tennessee Institute of Agriculture (UTIA)—East Tennessee Research and Education Center (ETREC) located at 35° 50′ 39″ N/83° 57′ 36″ W. Soils in the area of harvest were verified to be Inceptisols of the Emory Series with transitions from A horizon silt loams to B horizon silt clay loams taking place at approximately 25 cm. Five matched replicates of clonal individuals of *P. deltoides* and five *P. trichocarpa × deltoides* hybrid (10 trees total) were selected on the border of adjacent experimental blocks. Trees were harvested on August 14–15, 2014, nearing the end of their third season of growth. Each tree was felled using a chainsaw onto a plastic tarp. The stump, roots, and surrounding soil (approximately 100-cm diameter, 75-cm depth) were removed by a hydraulic tree spade and placed onto a separate tarp for dissection and processing. Thirty different plant-associated habitat types were defined and processed as outlined below across the 10 trees (*N* = 300; Additional file 1: Table S1 and Figure S1). Sample processing took place in both the field and laboratory. Field processed samples (e.g., soils, leaf swabs) were transported on blue ice and frozen at − 80 °C on the same day. Laboratory processed samples were stored in a 4 °C cold room until processing was completed as below.

### Host niche definitions and sample preparation

Root samples were extracted from shallow (0–30 cm) and deep (30–75 cm) depths of each tree’s root ball and stored at 4 °C until processed (within 4 days). Bulk soil was sampled from the same depth interval from the edge of the excavation hole, placed on ice and frozen at − 80 °C in the laboratory the same day until DNA was extracted. In the laboratory, shallow and deep roots were washed three times with 200 mL of 0.1% sterile Tween 20 and then separated by diameter classes into fine (< 2 mm) and coarse (~ 5–20 mm—termed secondary throughout the remainder of the text) roots. Soil attached to shallow and deep roots (referred to as shallow and deep rhizosphere habitats in the remainder of the text) was pelleted by centrifugation in 50-ml tubes and then frozen at − 80 °C until DNA was extracted. These root classes were then surface-sterilized as described previously [21, 22]. Structural roots (> 5 cm) from the two depths were also collected and processed identically to stem samples (described below). All root endosphere samples were verified as surface-sterile by streaking subsampled material across an R2A agar plate and incubating overnight at room temperature to check for the appearance of colonies. Samples with colonies present had this sterilization procedure repeated. Given our root sterilization procedure used sodium hypochlorite which has been shown to remove ~ 98% of microbes on the exterior of roots [31], we were unable to characterize the rhizoplane-associated microbial community.

Three stem sections from each annual growth increment, as identified by successive terminal bud scars, were collected and separated in the field, transported on ice, and then stored in a cold room at 4 °C until processed (within 10 days). In the laboratory, each stem and structural root section sample was wiped down with sterile 0.1% Tween 20 solution. Samples from each growth year (1, 2, and 3) were then dissected into three habitat categories: outer stem layer (i.e., bark, cambium, and phloem tissue), living developing xylem, and mature xylem tissue and preserved at − 80 °C until DNA extraction.

Leaf samples were collected from terminal (developing leaves, LPI 2–4) and sub-terminal (mature leaves, LPI 7–10) along multiple branches. The top surfaces (developing and mature upper phyllosphere) and bottom surfaces (developing and mature lower phyllosphere) of each leaf sample were then separately swabbed in the field with wooden applicators moistened with sterile 0.1% Tween 20, and swabs frozen at − 80 °C upon arrival to the laboratory, while leaves were stored at 4 °C until processing (within 6 days). Leaf and petioles were then separated and washed (developing whole leaf wash and mature whole leaf wash) and surface-sterilized (developing and mature leaf endosphere, developing and mature petiole endosphere) as described above for roots (Additional file 1: Table S1) and frozen at − 80 °C until DNA extraction. Due to storage time differences (i.e., frozen the day of sampling versus stored at 4 °C for several days prior to dissection or processing), we compared mean differences between significantly different leaf habitat comparisons (e.g., developing whole leaf phyllosphere [DWL, leaf phyllosphere washes up to 6-days storage] versus upper phyllosphere developing [UPD, leaf swabs frozen at day 0]) for alpha diversity ANOVAs and beta diversity (NMDS scores) ANOVAs. Leaf habitats differed, and had similar mean differences, between those that were sampled in the same timeframe and those sampled at different timeframes (e.g., bacterial diversity DWL vs UPD and DWL vs LEM mean difference = 0.23, *p* = 0.02). Therefore, storage time differences likely did not significantly alter our results.

### DNA extractions and Illumina MiSeq sequencing

All plant tissues (i.e., roots, stems, and leaves) were cut into fine pieces (< 5 mm) prior to DNA extraction. Rhizosphere samples, whole-leaf washes, and upper and lower phyllosphere samples were centrifuged at 10,000 rcf for 10 min, and the supernatant was removed. These samples and bulk soil samples had 250 mg of material extracted using the MoBio PowerSoil DNA Isolation Kit (MoBio Laboratories, Inc., Carlsbad, CA, USA). All other tissue types had 50 mg of tissue per extraction and were bead-beaten for 3 min in frozen (liquid nitrogen) blocks using one 5-mm steal bead per extraction (Qiagen, Venlo, the Netherlands). Following these steps, pulverized tissue was extracted using the MoBio PowerPlant Pro DNA Isolation Kit (MoBio Laboratories, Inc., Carlsbad, CA, USA). Stem tissue samples had two replicate extractions per sample to achieve sufficient DNA yields. All extractions were quantified on a NanoDrop 1000 spectrophotometer (NanoDrop Products, Wilmington, DE, USA). Aboveground tissues (i.e., leaves and stems) were also purified and concentrated using Zymo DNA Clean and Concentrator-5 Kit (Zymo Research Corporation, Irvine, CA, USA) and quantified again prior to PCRs.

We used a two-step PCR approach to barcode tag templates with frameshifting nucleotide primers [32] with the following modifications. Forward and reverse primer mixtures were modified to maximize phylogenetic coverage of archaea, bacteria, and fungi (Additional file 1: Table S2), thus allowing full and simultaneous assessment of bacteria, fungi, and archaea due to the increased coverage of our primer sets. Primers for tagging bacterial amplicons were a mixture of 9 forward and 6 reverse 515F and 806R primers for the 16S rRNA V4 gene region at equal concentrations (0.5 μM; Additional file 1: Table S2). Primers for tagging fungal ITS2 rRNA region included a mixture of 11 forward and 7 reverse primers at equal concentration (0.5 μM; Additional file 1: Table S2). To inhibit plant material amplification, we added a mixture of peptide nucleotide acid (PNA) blockers oligos (PNA Bio Inc., Thousand Oaks, CA, USA) targeted at plant mitochondrial and chloroplast 16S rRNA genes and plant 5.8S nuclear rRNA gene upstream of ITS2 region primers in fungal PCRs (see Lundberg et al. [32]; Additional file 1: Table S2 and Figure S2). The mitochondrial PNA of Lundberg et al. [32] was adjusted for a 1 bp mismatch in *Populus*, whereas the nuclear 5.8S PNA was custom-designed for this study. Thermal cycler conditions for the primary PCRs for soils were 5 cycles of 95 °C for 1 min, 50 °C for 2 min, and 72 °C for 1 min. Primary PCR conditions for plant tissues were 5 cycles of 95 °C for 1 min, 78 °C for 5 s, 50 °C for 2 min, and 72 °C for 1 min. Primary PCR products were cleaned with 17 μL of Agencourt AMPure beads and eluted in 21 μL of nuclease-free water. Secondary PCRs had purified DNA tagged with barcoded reverse primers and forward primers (Additional file 1: Table S2) in the 50 μL reaction, except with 20 μL of purified DNA from primary PCRs. Thermal cycler conditions for secondary soil PCRs consisted of denaturation at 95 °C for 45 s followed by 32 cycles of 95 °C for 15 s, annealing at 60 °C for 30 s, 72 °C for 30 s, and final extension at 72 °C for 30 s. Secondary PCRs for plant tissue consisted of denaturation at 95 °C for 45 s, followed by 32 cycles of 95 °C for 15 s, 78 °C for 5 s, with remaining cycle parameters the same as with soil secondary PCRs.

After PCRs, experimental units were pooled based on agarose gel band intensity and purified with Agencourt AMPure XP beads (beads to DNA, 0.7 to 1 ratio; Beckman Coulter Inc., Pasadena, CA, USA). Illumina MiSeq sequencing was carried out using a 9 pM amplicon concentration including a 15% PhiX spike with 500 (v. 2; 2 × 250) cycles.

### Illumina MiSeq sequence processing

Paired-end sequences (.fastq) were joined and demultiplexed using QIIME default settings except a Phred quality threshold of Q20 [33]. Forward and reverse primers were then removed using the cutadapt program [34]. For ITS2 sequences, reads were truncated to 200 bp and any sequences less than 200 bp were filtered. Both 16S and ITS2 sequences were quality-filtered (fastq_maxee = 0.5), derepelicated, and had singletons removed in USEARCH [35]. Operational taxonomic units (OTUs) were then clustered at 97% similarity after chimeras were removed in USEARCH [35]. Lastly, using QIIME [33], OTUs were classified using BLAST with the Greengenes (V13.8) and UNITE reference databases (V7.1; [36] for archaeal/bacterial and fungal communities, respectively). Contaminant sequences that were unclassified at domain (bacteria/archaea) or kingdom (fungi), mitochondria, chloroplasts, plant, and protista, were filtered. Complete datasets across habitat comparisons were rarefied at 1000 sequences for bacteria and 2000 for fungi to minimize sample loss (see rarefaction curves—Additional file 1: Figures S3–S4). The final full community dataset had 7458 OTUs and 269,000 sequences for bacteria and 9277 OTUs and 546,000 sequences for fungi. After the full dataset was analyzed, leaf, stem, root, and soil compartments were separated to examine differences within these compartments and each rarefied separately to maximize sequence number and minimize sample loss. Leaf, stem, and root samples were rarefied at 500 sequences for bacteria and 1000 sequences for fungi. Soil samples were rarefied at 35,000 for bacteria and 5000 sequences for fungi. OTU diversity was calculated in QIIME as the complement of Simpson’s Diversity (1 − *D* = 1 − *Σp*_i_^2^) with *p*_i_ representing the frequency of each OTU within a sample.

### Statistical analysis

We determined if the relative abundance of dominant fungal pathogens differed across leaf tissue habitats and genotypes (OTUs identified as *Mycosphaerella/Septoria* sp. and *Marssonina brunnea*), and whether dominant (≥ 0.1%) archaeal/bacterial and fungal phyla differed across broad habitat categories (i.e., leaf, stem, root, and soil), and between genotypes using two-way ANOVA models. We also used two-way ANOVAs to test if both archaeal/bacterial and fungal OTU diversity differed across habitats and between genotypes. Microbial diversity data was arc-sine transformed prior to ANOVAs. Since some phyla’s relative abundance was skewed, we used log_10_-transformed data to meet assumptions of normality prior to statistical analysis. Since multiple tests were run, each type 1 error rate for each ANOVA model was FDR-corrected for multiple comparisons. ANOVA models were performed in *R* (*aov* function, R Project for Statistical Computing, Vienna, Austria).

Microbial community composition was assessed by computing Bray–Curtis dissimilarity matrices and then visualized using non-metric dimensional scaling (NMDS) ordinations to visualize compositional differences. To test whether habitat, genotype, or their interaction had a significant effect on community composition, a permutational multivariate ANOVA (perMANOVA; [37]) with 10,000 permutations was calculated. NMDS and perMANOVA models were performed in Primer-E (Quest Research Limited, New Zealand). We also calculated perMANOVA pairwise comparisons within habitats and genotypes for leaf, stem, root, and soil communities separately for bacteria and fungi (*pairwise.perm.manova* in package RVAideMemoire; [38]). Lastly, we performed an indicator species analysis [39] using OTU abundance data to determine which OTUs occurred more frequently between habitats (i.e., leaf, stem, root, soil), genotype for all habitats (DD vs. TD), and genotype within a habitat (e.g., leaf DD vs. leaf TD) for bacterial and fungal communities (*multipatt* function in indicspecies package; [39]). After indicator OTUs were detected, an FDR correction was applied for post hoc multiple comparisons of statistical significance.

We used FUNGuild [40] to classify each OTU into an ecological guild to determine if fungal functional groups differed in relative abundance between genotypes within each broad habitat category (leaf, stem, root, soil). OTUs identified to a guild with a confidence ranking of “highly probable” or “probable” were retained in our analysis, whereas those with “possible” were considered unclassified. Furthermore, OTUs designated in more than one guild, with confidence, were placed in a “> 1 guild” category, but we do not report any results on this group of fungi. Undefined guilds, such as undefined pathogens, refer to pathogens not specific to fungi, plants, or animals, and undefined saprotrophs refer to saprotrophs not specific to wood, plant, or litter soil. A one-way ANOVA model was used to determine if dominant guilds within a habitat differed between plant genotypes. In this analysis, we included animal, plant, and undefined pathogens; soil, wood, and undefined saprotrophs; and fungal parasites, endophytes, arbuscular mycorrhizae, and ectomycorrhizae. Ericoid mycorrhizae were rarely detected in our dataset (i.e., present at low abundance within eight samples across all habitats); therefore, we did not include this guild in our analysis.

## Results

### Microbial community composition shifts across habitat and tree genotype

**Fig. 1:**
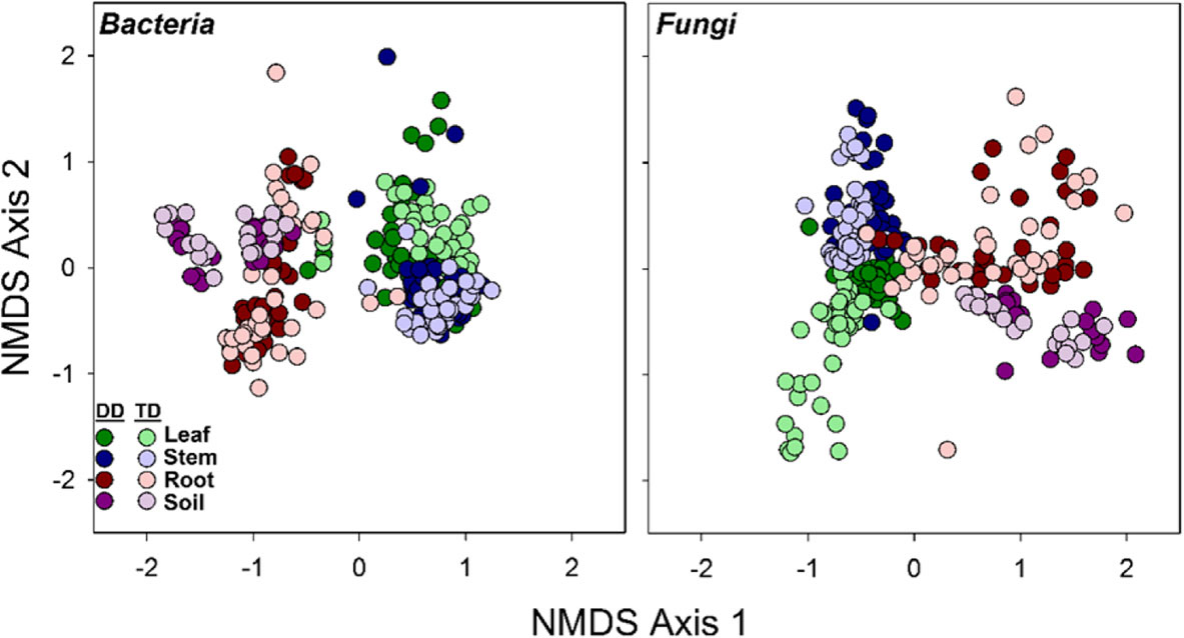
NMDS ordinations of both archaeal/bacterial and fungal communities across the four broad habitat classifications (leaves, stems, roots, soil) and genotypes (*P. deltoides*, *P. trichocarpa × deltoides* hybrid). Darker colors represent *P. deltoides* (DD) samples, whereas respective light colors represent hybrid samples (TD). Based on perMANOVA results, habitat was more influential for archaeal/bacterial and fungal community composition than genotype (Table 1)

**Fig. 2:**
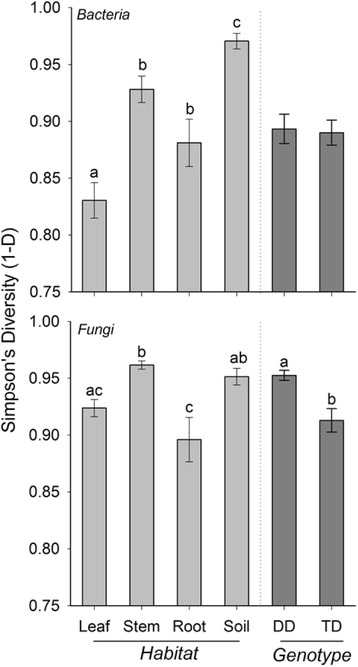
Simpson’s (1 − *D*) diversity across broad habitats (leaf, stem, root, and soil—denoted in light gray) and genotypes (DD = *P. deltoides*, TD = *P. trichocarpa × deltoides*—denoted in dark gray) for both archaeal/bacterial and fungal communities. For both archaea/bacteria and fungi, diversity differed across habitats (*p* ≤ 0.01). Fungal diversity differed between genotypes (*p* ≤ 0.01). Letters denote significant differences based on Tukey’s HSD post hoc comparison tests. Diversity was arcsine square root transformed prior to analysis, but raw data average and standard errors per habitat and genotype are shown

Across the four broad habitats sampled (i.e., leaf, stem, root, soil), we found significant differences in both archaeal/bacterial (*R*^2^ = 0.30) and fungal (*R*^2^ = 0.24) community composition (Fig. 1, Table 1). A small amount of variation in community composition was also explained by genotype (bacterial *R*^2^ = 0.02, fungal *R*^2^ = 0.03) and the habitat × genotype interaction (bacterial *R*^2^ = 0.04, fungal *R*^2^ = 0.08, Table 1). Archaeal/bacterial alpha diversity was greatest in soil habitats and lowest in leaf habitats (Fig. 2). Stem and root had similar bacterial alpha diversity estimates (*p* = 0.25). Fungal alpha diversity was greater in stems than in leaf or root habitats (*p* ≤ 0.01), whereas fungal alpha diversity was also greater in soils than roots (Tukey’s HSD, *p* = 0.05; Fig. 2). Archaeal/bacterial alpha diversity did not differ between *Populus* genotypes, but we found significant differences in alpha fungal diversity between *Populus* genotypes. Fungal diversity was greater in *P. deltoides* than in the hybrid (Fig. 2).

**Table 1:** Permutational multivariate ANOVA results with Bray–Curtis distance matrices implemented to partition sources of variation in this study (habitat, genotype, interaction between habitat and genotype (H × G)) for both archaeal/bacterial and fungal communities. All samples were included therefore the main effect of habitat represents the broad categories of leaves, stems, roots, and soils. Statistical significance (P(perm)) computed based on sequential sums of squares from 9999 permutations

Community	Source of variation	SS	MS	*R* ^2^	Pseudo-*F*	P(perm)
Bacteria	Habitat	307,710	102,570	0.30	40.3	0.0001
	Genotype	18,469	18,469	0.02	7.3	0.0001
	Interaction	41,533	13,844	0.04	5.4	0.0001
	Residuals	663,810	2543.3	0.64		
	Total	1,036,500		1		
Fungi	Habitat	246,890	82,295	0.24	32.2	0.0001
	Genotype	26,376	26,376	0.03	10.3	0.0001
	Interaction	79,953	26,651	0.08	10.4	0.0001
	Residuals	677,060	2554.9	0.65		
	Total	1,043,500		1

**Fig. 3:**
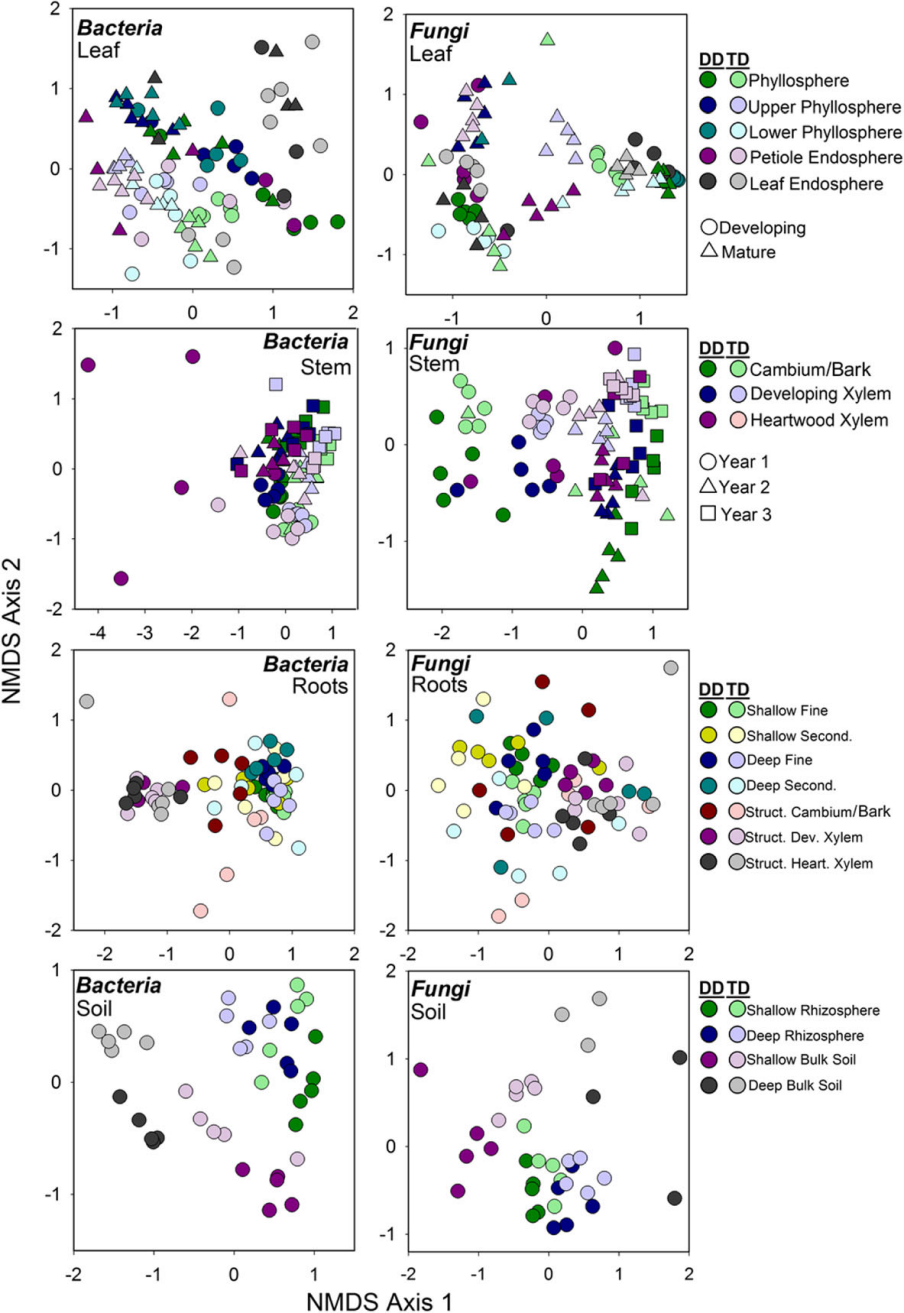
NMDS ordination of both archaeal/bacterial and fungal communities across all 30 leaf, stem, root, and soil niches and *Populus* genotypes (*P. deltoides*, *P. trichocarpa × deltoides* hybrid). Darker colors represent *P. deltoides* (DD) samples whereas respective light colors represent hybrid samples (TD). For leaf communities: circles = developing leaf samples and triangles = mature leaf samples. For stem communities: circles = year 1, triangles = year 2, and squares = year 3 samples. Niche was most influential for archaeal/bacterial communities for leaves and stems, whereas genotype was most influential for fungal communities in leaves only. Niche was more influential for fungi in stems (Table 2). For roots and soils, in both archaeal/bacterial and fungal communities, niche was more influential than genotype (Additional file 1: Table S12)

Within each broad habitat, the main effect of finer-scale habitat (within leaf, stem, roots, soils separately referred to as niche in remainder of text; Fig. 3) explained more variation than genotype or their interaction (Table 2), except for leaf fungal communities. Genotype explained more variation in leaf fungal community composition than niche (*R*^2^ = 0.21). Furthermore, across leaf, root, and soil communities, niche was more influential for archaeal/bacterial composition than fungal, whereas in stem communities, niche explained more variation in fungal communities compared to archaeal/bacterial communities (Table 2). The main effect of genotype generally explained similar amounts of variation for archaea/bacteria and fungi across specific niches, except for leaf communities (Table 2). Archaeal/bacterial diversity also differed among niches within each broad tissue/habitat type (e.g., whole-leaf washes had lower diversity than upper phyllosphere in developing tissues), but did not differ between genotypes within each niche across the broad tissue/habitat types (Additional file 1: Tables S5–S8). Fungal diversity differed between niches within broad habitat types, except roots. Further, niches within leaves, stems, and root communities differed in fungal diversity between genotype (*p* ≤ 0.04; Additional file 1: Tables S9–S11), where *P. deltoides* had greater fungal diversity, on average, compared to the hybrid (Fig. 2).

**Table 2:** Permutational multivariate ANOVA results with Bray–Curtis distance matrices implemented to partition sources of variation in this study (niche, genotype, interaction between niche and genotype (N × G)) for both archaeal/bacterial and fungal communities. Leaves, stems, roots, and soil communities were analyzed separately; therefore, habitat effects refer to finer-scale niches within these respective broad habitat categories. Statistical significance (P(perm)) was computed based on sequential sums of squares from 9999 permutations

Community	Habitat	Source of variation	SS	MS	*R* ^2^	Pseudo-*F*	P(perm)
Bacteria	Leaves	Niche	78,980	8775.6	0.29	5.0	0.0001
		Genotype	26,849	26,849	0.10	15.2	0.0001
		N × G	37,557	4173	0.14	2.4	0.0001
		Residuals	125,080	1761.7	0.46		
		Total	272,940		1.00		
Fungi	Leaves	Niche	48,697	6087.1	0.21	5.6	0.0001
		Genotype	72,650	72,650	0.32	66.9	0.0001
		N × G	29,836	3729.5	0.13	3.4	0.0001
		Residuals	73,840	1085.9	0.32		
		Total	229,760		1.00		
Bacteria	Stem	Niche	58,966	7370.8	0.31	5.4	0.0001
		Genotype	19,362	19,362	0.10	14.3	0.0001
		N × G	20,049	2506.2	0.10	1.8	0.0001
		Residuals	92,356	1358.2	0.48		
		Total	191,000		1.00		
Fungi	Stem	Niche	90,054	11,257	0.36	7.0	0.0001
		Genotype	22,613	22,613	0.09	14.0	0.0001
		N × G	21,776	2722	0.09	1.7	0.0001
		Residuals	114,460	1612.1	0.46		
		Total	249,640		1.00		
Bacteria	Roots	Niche	81,253	13,542	0.39	6.9	0.0001
		Genotype	6752.2	6752.2	0.03	3.4	0.0001
		N × G	13,966	2327.6	0.07	1.2	0.0213
		Residuals	106,410	1970.6	0.51		
		Total	210,180		1.00		
Fungi	Roots	Niche	53,637	8939.5	0.20	2.8	0.0001
		Genotype	9286	9286	0.04	2.9	0.0001
		N × G	23,063	3843.8	0.09	1.2	0.0183
		Residuals	177,350	3167	0.67		
		Total	263,340		1.00		
Bacteria	Soil	Niche	41,055	13,685	0.51	18.9	0.0001
		Genotype	8807.4	8807.4	0.11	12.2	0.0001
		N × G	7020.7	2340.2	0.09	3.2	0.0001
		Residuals	23,182	724.5	0.29		
		Total	80,066		1.00		
Fungi	Soil	Niche	28,882	9627.3	0.31	5.9	0.0001
		Genotype	8470.1	8470.1	0.09	5.2	0.0001
		N × G	8920.8	2973.6	0.10	1.8	0.0001
		Residuals	45,882	1638.6	0.50		
		Total	92,296		1.00		

### Phylum level differences across habitat and tree genotype

Twenty-one dominant (≥ 0.1% relative abundance) archaeal/bacterial phyla, and classes for Proteobacteria, were detected across this study (Additional file 1: Table S12). Twenty of these 21 dominant archaeal/bacterial phyla differed across broad habitats (i.e., leaves, stem, roots, and soil; *F*_3,267_ = 12.55, *p* ≤ 0.01, Fig. 3). Fusobacteria is the only dominant phyla that did not differ across these habitats (Additional file 1: Table S12). Crenarchaeota, Firmicutes, Nitrospirae, AD3, and WS3 had greater abundance in soils than in roots, stems, and leaves (Tukey’s HSD: *p* ≤ 0.01). The most common archaeal phyla identified, the Crenarchaeota, differed significantly across all tested habitats. The Crenarchaeota had 0.3% relative abundance in the leaves, 0.1% relative abundance in the stems, 0.2% abundance in the roots, and 3.0% relative abundance in the soil. Acidobacteria, Chloroflexi, Planctomycetes, Verrucomicrobia, and Deltaproteobacteria had the greatest abundance in soil versus other habitats, but also had greater abundance in roots than in stems and leaves (Tukey’s HSD: *p* ≤ 0.01). Gemmatimonadetes had the greatest abundance in soil, and root habitats had greater abundance compared to stem tissues (Tukey’s HSD: *p* ≤ 0.01). Bacteroidetes had the greatest abundance in roots and stems compared to leaves and soil habitats, whereas TM7 had the greatest abundance in root habitats compared to all other habitats (Tukey’s HSD: *p* ≤ 0.01). Actinobacteria and Armatimonadetes had greater abundance in soils, roots, and stems than in leaves, whereas TM6 had greater abundance in soils, roots, and leaves than in stem habitats (Tukey’s HSD: *p* ≤ 0.01). Phylum FBP had greatest abundance in stem tissues (Tukey’s HSD: *p* ≤ 0.01). Alphaproteobacteria also had the greatest abundance in stem tissues. Leaves were enriched in Alphaproteobacteria compared to roots and soil and in root tissues compared to soil habitats (Tukey’s HSD: *p* ≤ 0.03; Additional file 1: Table S12). Betaproteobacteria were most abundant in soils and roots than in leaves or stems. Leaves were enriched in Betaproteobacteria compared to stems (Tukey’s HSD: *p* ≤ 0.03). Gammaproteobacteria were most abundant in roots and leaves than in soils and stem habitats (Tukey’s HSD: p ≤ 0.01). Actinobacteria were more abundant in *P. deltoides*-associated tissue/habitats, whereas TM7 were more abundant in the hybrid (*p* ≤ 0.03).

**Fig. 4:**
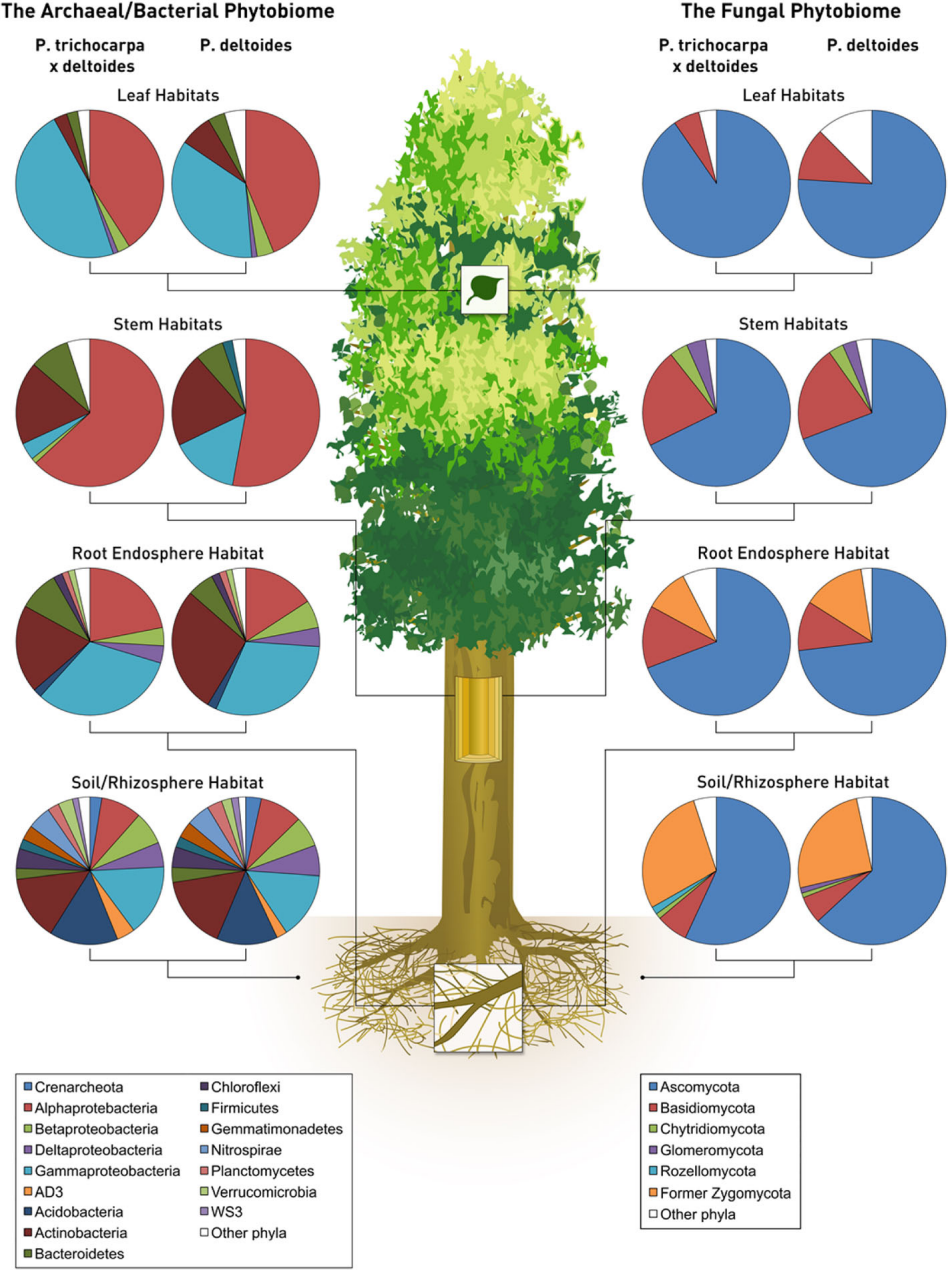
Relative abundance of the dominant (> 0.1%) archaeal/bacterial phyla—class for proteobacteria—and fungal phyla averaged across niches within the broad tree habitats of leaves, stems, roots, and soils within *Populus delotides* (DD) and *Populus trichocarpa × deltoides* (TD) hybrids

All six fungal phyla were found in this study (Fig. 4, Additional file 1: Table S12). Basidiomycota, Chytridiomycota, and Glomeromycota were most abundant in stem habitats (Tukey’s HSD: *p* ≤ 0.01). Ascomycota were most abundant in leaves and lowest in soils contrary to Rozellomycota and the former Zygomycota, which were most abundant in soils (Tukey’s HSD: *p* ≤ 0.01). No fungal phyla differed in abundance between tree genotypes.

### Functional fungal guild and OTU differences across tree genotype

Several functional guilds’ relative abundance differed between genotypes. Within soils, one functional guild differed between genotypes. Soil saprotrophs had greater relative abundance in the hybrid genotype compared to *P. deltoides* (*F*_1,39_ = 4.45, *p* = 0.04), but soil saprotrophs had, on average, low abundance (0.08%). In roots, undefined pathogens were greater in the hybrid genotype (*F*_1,63_ = 5.96, *p* = 0.02), but at very low abundance (undefined pathogens: 0% in *P. deltoides*, 0.03% in hybrids). In stems, low-abundance guilds, such as animal pathogens (*F*_1,85_ = 5.51, *p* = 0.02) and fungal parasites (*F*_1,85_ = 16.66, *p* < 0.001), were greater in hybrids compared to *P. deltoides* (0.1%, 0.4 vs. 0.03%, 0.03%, respectively), but abundant plant pathogens were approximately 2× greater in *P. deltoides* compared to the hybrid genotype (*F*_1,85_ = 16.20, *p* < 0.001; 18.2% mean relative abundance in *P. deltoides* vs. 8.9% in *P. trichocarpa × deltoides*). Leaves had greater animal pathogens (*F*_1,81_ = 4.08, *p* = 0.05), endophytes (*F*_1,81_ = 7.81, *p* = 0.007), and undefined saprotrophs in *P. deltoides* tissue (0.02%, 0.06%, 6.4%) compared to hybrid plants (0.01%, 0.02%, 1.6%, respectively). Interestingly, plant pathogen relative abundance did not differ between genotypes in leaf tissues (9.2% *P. deltoides*, 8.7% hybrids; *p* = 0.810).

**Fig. 5:**
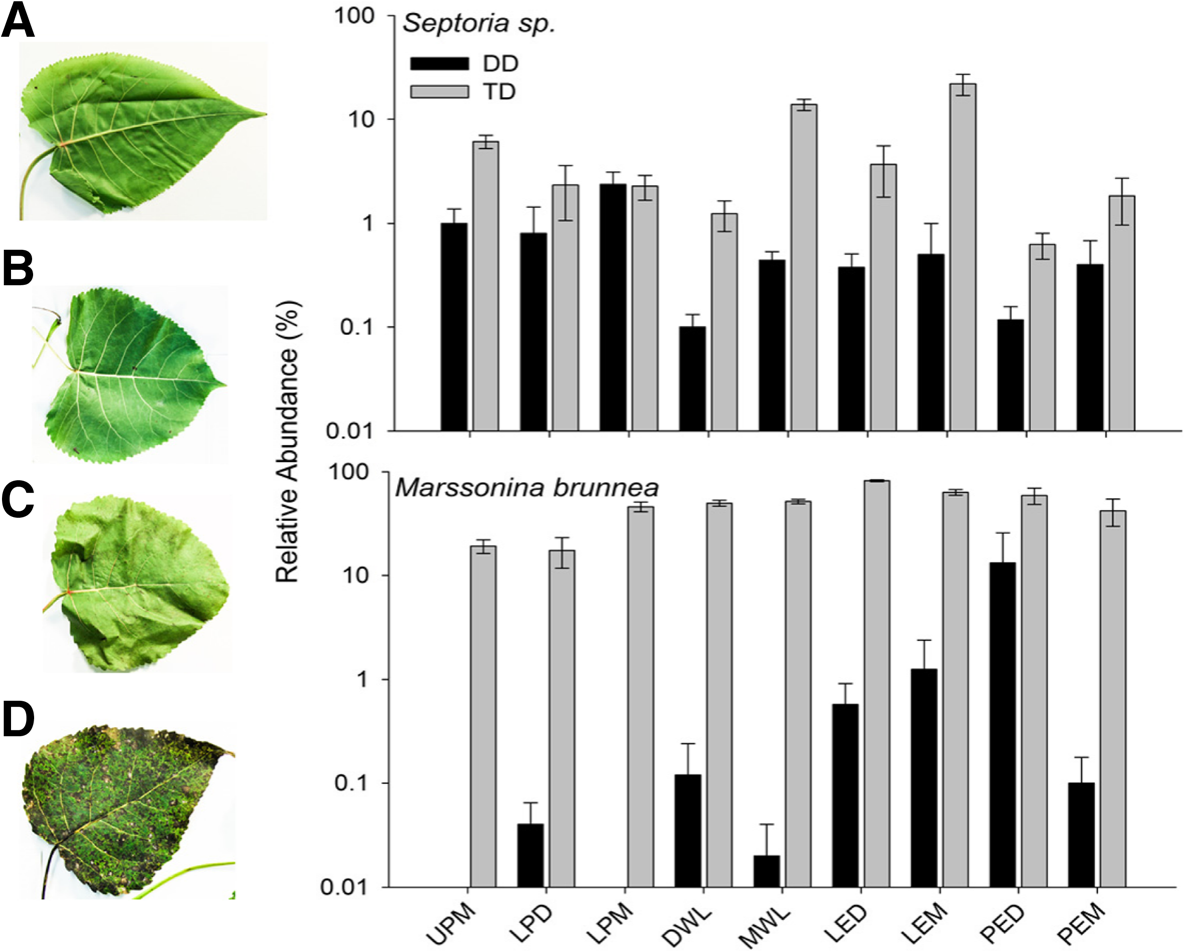
Log relative abundance of OTUs classified as the *Populus* leaf pathogens *Septoria* sp. and *Marssonina brunnea* across leaf niches within *P. deltoides* (DD) and *P. trichocarpa × deltoides* hybrids (TD). Bars represent means ± SE. Any bars missing indicate that OTU is absent from all samples within that habitat category. *Septoria sp.* and *Marssonina brunnea* relative abundance differed across habitats and genotypes (*p* ≤ 0.05). Representative of developing vs. mature leaves for *Populus* deltoides (**a** vs **b**) and TD hybrids (**c** vs **d**), respectively. The *X*-axis denotes leaf niche sampled. UPM upper phyllosphere mature, LPD lower phyllosphere developing, LPM lower phyllosphere mature, DWL whole leaf developing phyllosphere, MWL whole leaf mature phyllosphere, LED leaf endosphere developing, LEM leaf endosphere mature, PED petiole endosphere developing, PEM petiole endosphere mature

Several OTUs were detected for both bacteria and fungi that significantly differed across habitats and between genotypes (Table 3). Across broad habitat categories, there were four OTUs that were indicative of leaf habitats, specifically *Pseudomonas* sp. and OTUs with highest taxonomic affinity to Ascomycota (*p* ≤ 0.01). BLASTn confirmed these classifications and identified the Ascomycota OTUs as *Marssonina brunnea*. One fungal indicator was found for stem habitats, classified in Chytridiomycota using UNITE, but classified as unicellular algae in BLASTn, so this OTU may potentially be a contaminant. Three indicator taxa existed for root tissues—*Pseudomonas* sp., *Codineaopsis* sp., and an uncultured ascomycete (Table 3). The same two fungal OTUs (OTU 2, 14988), which were indicators for leaf tissue (*Marssonina brunnea*), were also indicators for the *P. trichocarpa × deltoides* hybrid across all broad habitat categories (relative abundance 7.1 and 13.4%, respectively; Table 3). Within leaf communities, several fungal OTUs were indicators for hybrid genotype tissues and were classified as *Marssonina brunnea* via BLASTn. Further, one fungal OTU, *Telletiopsis washingtonensis*, was an indicator for *P. deltoides* leaf tissue. Lastly, within stem communities, two bacteria OTUs—*Curtobacterium flaccumfaciens* and *Elsinoe banksiae*—were indicators for hybrid stem tissue (Table 2). The relative abundance of both *Septoria* sp. and *Marssonina brunnea*, common *Populus* pathogens, differed across leaf niches and genotypes (Additional file 1: Tables S3–S4). Notably, both potential fungal pathogens were significantly greater in relative abundance in hybrid ramets (Fig. 5).

**Table 3:** Indicator species analysis for bacterial and fungal OTUs across all samples (all samples community) and in leaf and stem communities. No indicator OTUs were detected for root or soil communities. Only dominant OTUs (≥ 1.0% relative abundance across samples) are given

Community	Treatment	OTU no.	DB classification	BLASTn classification	Identity percentage/E-value	Relative abundance
All samples	Leaf	6	*Pseudomonas sp.*	*Pseudomonas sp.*	100/3e−128	2.2
All samples	Leaf	14	*Pseudomonas sp.*	*Pseudomonas oryzihabitans* strain*	100/3e−128	1.9
All samples	Leaf	2	Ascomycota	*Marssonina brunnea*	100/6e−99	4.3
All samples	Leaf	14,988	Ascomycota	*Marssonina brunnea*	100/5e−95	2.2
All samples	Stem	16	Chytridiomycota	*Trebouxia impressa*	100/6e−99	1.3
All samples	Root	11,331	*Pseudomonas sp.*	*Pseudomonas sp.*	98/1e−121	1.3
All samples	Root	10,451	*Codinaeopsis sp.*	Codinaeopsis sp.**	99/2e−94	1.6
All samples	Root	42	Ascomycota	Uncultured fungus	98/3e−72	1.1
All samples	TD	2	Ascomycota	*Marssonina brunnea*	100/6e−99	4.3
All samples	TD	14,988	Ascomycota	*Marssonina brunnea*	100/5e−95	2.2
Leaf	DD	66	Exobasidiomycetes	*Telletiopsis washingtonensis*	100/6e−99	1.1
Leaf	TD	14,988	Ascomycota	*Marssonina brunnea*	100/5e−95	13.4
Leaf	TD	2	Ascomycota	*Marssonina brunnea*	100/6e−99	7.1
Leaf	TD	6721	Ascomycota	*Marssonina brunnea*	99/1e−90	1.7
Leaf	TD	2744	Ascomycota	*Marssonina brunnea*	99/7e−89	1.1
Leaf	TD	3701	Ascomycota	*Marssonina brunnea*	98/7e−89	1.0
Leaf	TD	19,038	Ascomycota	*Marssonina brunnea*	100/8e−93	1.0
Stem	TD	151	Microbacteriaceae	*Curtobacterium flaccumfaciens* strain	100/3e−128	1.6
Stem	TD	14,143	*Sphaceloma protearum*	*Elsinoe banksiae*	96/7e−84	2.6

*Representative sequence also had significant alignments with *Pseudomonas psychrotolerans* strains (identity percentage = 100%, E-value = 3e−128)

**Representative sequence also had significant alignments with *Codinaea acacieae* and *Fusarium* sp. However, all other high-quality hits were either with *Codinaeopsis* sp. or Chaetosphaeriales, the order *Codinaeopsis* belongs in (identity percentage = 99%, E-value = 2e−94)

## Discussion

This study demonstrates that the *Populus* microbiome significantly differs across the soil-root-stem-leaf landscape (Additional file 1: Table S12) and at a finer scale (within each of these niches; Fig. 3). Both archaeal/bacterial and fungal community composition shifted more so across habitats than between tree genotype when considering broad habitat classifications (i.e., soils, roots, stems, and leaves; Table 1) indicating environmental filtering (e.g., tissue specific filters) as a strong selective force for microbial communities across these environments. However, the fungal microbiome within leaf habitats varied more so between genotypes compared to habitat (Table 2), likely influenced by the dominance of two fungal pathogens, *Marssonina brunnea* and *Septoria sp.*, within leaves (Fig. 5 and Table 3). These pathogens likely impacted turnover of microbial populations within the susceptible hybrid ramets. Bacterial diversity was greater in soils relative to roots, and aboveground habitats, but contrary to this, fungal diversity was similar between soils, stems, and leaves, whereas stem fungi had greater diversity compared to leaves and roots (Fig. 2). These results suggest not only that niche-based processes (i.e., habitat selection) largely drive both archaeal/bacterial and fungal community assembly across plant tissues, but also that specific mechanisms of assembly (e.g., niche partitioning, life history strategies) differ for archaea/bacteria and fungi across the *Populus* environment. However, due to amplification issues with specific tissues (i.e., rarefying at 500 sequences for bacterial communities), conclusions regarding microbial diversity may be limited in this particularly study and warrant further validation.

### Habitat selection effects

Assembly of plant-associated microbial communities may be driven by niche-based processes, specifically plant genetic factors [41], acquisition via tissue-level selection, or stochasticity [42]. We observed significant differences in microbial diversity and community composition across broadly defined habitats (Figs. 1, 2, 3, and 4), and within these habitat categories (Additional file 1: Tables S5–S11). This agrees with our hypotheses that microbial communities would vary across the plant niches surveyed and is likely due to differences in regional species pools that colonize the various habitats (e.g., soil for roots, rainfall and aerial dispersal for leaves and stems) and niche partitioning as an outcome of microbial life history differences. The variation attributed to habitat, or plant tissue type, as a control on community composition indicates the strength of biotic (plant selection) or abiotic drivers of microbiome differentiation. Selection of microbial members across habitats are likely due to (1) interplay with *Populus* biochemical products [43], (2) mutualistic associations via plant growth-promoting microbes, or (3) large differences in abiotic factors such as nutrient availability and light exposure within aboveground tissues compared to belowground [6]. The latter may be especially relevant for the differences in archaeal/bacterial and fungal diversity across habitats. Fungal species, which are generally more tolerant to desiccation compared to bacteria, may proliferate under harsh environments (e.g., phyllospheres). Due to stress tolerance, or perhaps more overlap in fungal niche requirements, a greater degree of coexistence may exist for fungal communities within aboveground tissue [44].

Consistent with other studies, microbial diversity differed between plant-associated habitats, and common bacterial and fungal phyla were seen across each of the habitats that were broadly comparable to other plant hosts [7, 8, 45]. Leaves were primarily dominated by Alphaproteobacteria and Ascomycota, the latter in part likely due to the highly abundant *Marssonina brunnea* and *Septoria musiva*-like pathogens, both ascomycetes. Stem tissues were likewise dominated by these groups, but also were enriched in Actinobacteria and Basidiomycota, Chytridiomycota, and Glomeromycota (Additional file 1: Table S12). Gammaproteobacteria and Actinobacteria, as well as representatives of the former fungal Zygomycota, were most abundant in root tissues (Table 2). Many of these same taxa were reported in *Populus trichocarpa* roots as part of the *Populus* genome study [14]. Surprisingly, based on fungal guild designations, we found less than 2% of fungi classified as mycorrhizal (both arbuscular and ectomycorrhizae) across tree genotypes. This result is surprising as both AM and ECM fungi readily colonize poplar tree roots [46]. However, due to plant pathogens dominating plant tissues, albeit primarily stems and roots, their presence may have prevented significant recruitment of beneficial mycorrhizae. In addition, chemical cues, such as phenolic compound production, common in *Populus*, may trigger fungal pathogen growth at low concentrations [47, 48] and therefore cause significant species turnover in the microbiome.

Within some of the niches, there were indications that microbial function varied significantly across tissues and between tree genotype. For example, within *Populus deltoides* first-year heartwood xylem, there was a surprisingly large divergence from other similar stem niches (Fig. 3) that was driven by a large number of Firmicutes (~ 20%), of which 11% were from a single *Lactobacillus* classified OTU. Multiple studies have suggested that heartwood environments (especially wetwood characteristic of *Populus* trees) can turn anoxic and harbor organisms capable of fermentation, nitrogen fixation, and methanogenesis [49–52]. However, heartwood formation in these 3-year-old trees was likely incomplete as this event does not generally occur in *Populus* until years 3 to 5 depending on rate of growth [53, 54]. Our results suggest we may be observing the beginnings of this change and its effects within this understudied microbial niche.

### *Populus* genotype effects

Between *P. deltoides* and *P. trichocarpa × deltoides* genotypes, we observed significant differences in both fungal diversity and composition within the broad habitat categories that are likely driven by greater fungal pathogen abundances in the hybrid trees (Fig. 2, Fig. 5). While cursory examination of the site had revealed characteristic *Septoria* stem cankers on the trees prior to the study (C. Schadt, personal observation), the high pathogen load and co-occurrence of both *Septoria* and *Marssonina* OTU within the hybrid trees was surprising and not recognized prior to the molecular analyses as we had assumed the leaf spots were also caused by *Septoria*. *P. deltoides* are resistant to certain sympatric fungal pathogens due to coevolution in the Eastern USA [55], whereas the hybrid trees are susceptible due to lack of co-occurring pathogens in the Western USA [27]. Indeed, the severity of loss from *Septoria* stem cankers and premature defoliation from the *Marssonina* leaf spot are the principle reasons hybrid poplar trees have not been commercially viable in the Eastern USA versus the Western USA where hybrids are grown for the pulp and paper industry [56]. While these fungal pathogens cause leaf spots and stem cankers, our results also demonstrate that they inhabit soils surrounding the plants and colonize root tissue, although relative abundance is significantly lower (less than 0.1%) in these habitats. Fungal pathogens in hybrid trees invade host tissue and may outcompete other fungal species leading to lower diversity in the hybrid fungal microbiome. This pattern is evident in the leaf tissues of the hybrid trees where *Marssonina brunnea* OTUs have a greater abundance. However, it is noteworthy that *Septoria sp.* were also present within tissues of both *P. deltoides* and the hybrid trees (Fig. 5) but only manifested disease symptoms in the hybrid. In the hybrid leaf tissues, *Septoria* OTUs were also at a much lower abundance than *Marssonina* OTUs (Fig. 2) suggesting that these pathogens are both able to colonize and coexist, but *Marssonina* may have ecological strategies which allow it to more readily colonize the leaf habitats and proliferate.

## Conclusions

The *Populus* woody plant system provides a relevant model to examine how microbial communities vary across tissue level niches. Overall, this study demonstrates how niche-based processes, such as environmental filtering or biotic interactions, drive microbiome composition and diversity within tree species. Further, this study indicates the potential importance of microbe-microbe interactions in microbial community composition as indicated by the presence of fungal pathogens which may alter the microorganisms inhabiting the hybrid *Populus* trees. However, while we suspect that the pathogens are playing a disproportionate role in structuring these communities, future studies will be needed to more carefully address this hypothesis using closely related pathogen-resistant and susceptible *Populus* genotypes.

## Additional files

Additional file 1: Table S1. . Sampling niches across the broad habitats and the three letter unique code for each niche.** Table S2.** Primer mixtures and PNA PCR blockers used in this study. Sequences in blue represent NextEra annealing sites, black represents the Molecular Identifier Tag including frameshifts, green represents linker adaptors, and red represents PCR primers.** Table S3.** Two-way ANOVA (habitat × genotype) *p* values for Tukey’s HSD post hoc pairwise comparisons test in *Septoria sp.* relative abundance differences across leaf habitats. Models indicate that *Septoria sp.* differed across habitats (*F* = 9.34, *p* ≤ 0.01) and tree genotypes (*F* = 56.34, *p* ≤ 0.01). **Table S4.** Two-way ANOVA (habitat × genotype) *p* values for Tukey’s HSD post hoc pairwise comparisons test in *Marssonina brunnea.* Relative abundance differences across leaf habitats. Models indicate that *Marssonina* differed across habitats (*F* = 6.40, *p* ≤ 0.01) and tree genotypes (*F* = 590.95, *p* ≤ 0.01). **Table S5.** Two-way ANOVA (habitat × genotype) *p* values for pairwise comparisons in bacterial diversity across leaf niches. Two-way ANOVA models indicate that bacterial diversity differed within habitats (*F* = 2.53, *p* = 0.013), but not between tree genotypes (*F* = 0.003, *p* = 0.958). **Table S6.** Two-way ANOVA (habitat × genotype) *p* values for pairwise comparisons in bacterial diversity across stem niches. Two-way ANOVA models indicate that bacterial diversity differed within habitats (*F* = 2.984, *p* = 0.006), but not between tree genotypes (*F* = 1.386, *p* = 0.243). **Table S7.** Two-way ANOVA (habitat × genotype) *p* values for pairwise comparisons in bacterial diversity across root niches. Two-way ANOVA models indicate that bacterial diversity differed within habitats (*F* = 11.474, *p* < 0.001), but not between tree genotypes (*F* = 0.987, *p* = 0.324). **Table S8.** Two-way ANOVA (habitat × genotype) *p* values for pairwise comparisons in bacterial diversity across soil niches. Two-way ANOVA models indicate that bacterial diversity differed within habitats (*F* = 7.821, *p* < 0.001), but not between tree genotypes (*F* = 0.297, *p* = 0.589). **Table S9. **Two-way ANOVA (habitat × genotype) *p* values for pairwise comparisons in fungal diversity across leaf niches. Two-way ANOVA models indicate that fungal diversity differed within habitats (*F* = 8.198, *p* < 0.001), and between tree genotypes (*F* = 86.509, *p* < 0.001). **Table S10.** Two-way ANOVA (habitat × genotype) *p* values for pairwise comparisons in fungal diversity across stem niches. Two-way ANOVA models indicate that fungal diversity differed within habitats (*F* = 4.568, *p* < 0.001), and between tree genotypes (*F* = 6.127, *p* = 0.015). **Table S11.** Two-way ANOVA (habitat × genotype) *p* values for pairwise comparisons in fungal diversity across soil niches. Two-way ANOVA models indicate that fungal diversity differed within habitats (*F* = 6.026, *p* = 0.002), but not between tree genotypes (*F* = 0.036, *p* = 0.851). **Table S12. **Relative abundance of dominant (≥ 0.1%) archaeal/bacterial and fungal phyla and class for Proteobacteria across broad habitat categories and genotypes (mean ± SE). Two-way ANOVA models indicated all bacterial and fungal phyla, except Fusobacteria, differed across habitat (*p* ≤ 0.01) whereas two bacterial phyla differed between genotypes (*p* ≤ 0.03) as denoted by bolded lettering. Letters denote Tukey’s HSD significant differences for main effects of habitat and genotype.** Figure S1. **Sampling schema for 30 plant niches. Each niche was sampled from five replicate *Populus deltoides* clones and *P. trichocarpa × deltoides* hybrid clones, totaling 300 microbiome samples. **Figure S2.** Performance of plant nuclear 5.8S rRNA gene targeted peptide nucleic acid (PNA) blocker in select fungal ITS2 amplicon libraries. We used two different tissue types including fine root endosphere (SFR), developing leaf endosphere (LED), as well as rhizosphere soils. These are tested on samples originating from *Populus deltoides* (DD1) and a *P. trichocarpa × deltoides* hybrid (TD1). Samples with PNA blockers added are indicated by _PNA at the end of name.** Figure S3. **Rarefaction curves for bacteria across broad habitat classifications (leaves—red, stems—green, roots—blue, soil—orange) at 1000 sequences per sample depth. **Figure S4.** Rarefaction curves for fungi across broad habitat classifications (leaves—red, stems—green, roots—blue, soil—orange) at 2000 sequences per sample depth. (DOCX 3250 kb)
