# Supplementary material for: The Populus holobiont: dissecting the effects of plant niches and genotype on the microbiome
Source: Microbiome. 2018 Feb 12;6:31. doi: 10.1186/s40168-018-0413-8 (PMC5810025; doi:10.1186/s40168-018-0413-8)
Supplement: Supplementary file 1 — . Sampling niches across the broad habitats and the three letter unique code for each niche. Table S2. Primer mixtures and PNA PCR blockers used in this study. Sequences in blue represent NextEra annealing sites, black represents the Molecular Identifier Tag including frameshifts, green represents linker adaptors, and red represents PCR primers. Table S3. Two-way ANOVA (habitat × genotype) p values for Tukey’s HSD post hoc pairwise comparisons test in Septoria sp. relative abundance differences across leaf habitats. Models indicate that Septoria sp. differed across habitats (F = 9.34, p ≤ 0.01) and tree genotypes (F = 56.34, p ≤ 0.01). Table S4. Two-way ANOVA (habitat × genotype) p values for Tukey’s HSD post hoc pairwise comparisons test in Marssonina brunnea. Relative abundance differences across leaf habitats. Models indicate that Marssonina differed across habitats (F = 6.40, p ≤ 0.01) and tree genotypes (F = 590.95, p ≤ 0.01). Table S5. Two-way ANOVA (habitat × genotype) p values for pairwise comparisons in bacterial diversity across leaf niches. Two-way ANOVA models indicate that bacterial diversity differed within habitats (F = 2.53, p = 0.013), but not between tree genotypes (F = 0.003, p = 0.958). Table S6. Two-way ANOVA (habitat × genotype) p values for pairwise comparisons in bacterial diversity across stem niches. Two-way ANOVA models indicate that bacterial diversity differed within habitats (F = 2.984, p = 0.006), but not between tree genotypes (F = 1.386, p = 0.243). Table S7. Two-way ANOVA (habitat × genotype) p values for pairwise comparisons in bacterial diversity across root niches. Two-way ANOVA models indicate that bacterial diversity differed within habitats (F = 11.474, p < 0.001), but not between tree genotypes (F = 0.987, p = 0.324). Table S8. Two-way ANOVA (habitat × genotype) p values for pairwise comparisons in bacterial diversity across soil niches. Two-way ANOVA models indicate that bacterial diversity differed within habitats (F = 7.82 [file 40168_2018_413_MOESM1_ESM.docx]

**Additional file 1**

**Table S1:** Sampling niches across the broad habitats and the three letter unique code for each niche.

| **Sample niche** | **Broad habitat** | **Code** |
| --- | --- | --- |
| Upper phyllosphere developing | Leaves | UPD |
| Lower phyllosphere developing | Leaves | LPD |
| Whole leaf phyllosphere developing | Leaves | DWL |
| Leaf endosphere developing | Leaves | LED |
| Petiole endosphere developing | Leaves | PED |
| Upper phyllosphere mature | Leaves | UPM |
| Lower phyllosphere mature | Leaves | LPM |
| Whole leaf phyllosphere mature | Leaves | MWL |
| Leaf endosphere mature | Leaves | LEM |
| Petiole endosphere mature | Leaves | PEM |
| 1^st^ year stem – heartwood xylem | Stems | 1HX |
| 1^st^ year stem – developing xylem | Stems | 1DX |
| 1^st^ year stem – cambium/phloem/bark | Stems | 1CB |
| 2^nd^ year stem – heartwood xylem | Stems | 2HX |
| 2^nd^ year stem – developing xylem | Stems | 2DX |
| 2^nd^ year stem – cambium/phloem/bark | Stems | 2CB |
| 3^rd^ year stem – heartwood xylem | Stems | 3HX |
| 3^rd^ year stem – developing xylem | Stems | 3DX |
| 3^rd^ year stem – cambium/phloem/bark | Stems | 3CB |
| Shallow fine roots | Roots | SFR |
| Deep fine roots | Roots | DFR |
| Shallow secondary roots | Roots | SSR |
| Deep secondary roots | Roots | DSR |
| Structural root – heartwood xylem | Roots | SHX |
| Structural root – developing xylem | Roots | SDX |
| Structural root – cambium/phloem/bark | Roots | SCB |
| Shallow rhizosphere | Soil | SRZ |
| Deep rhizosphere | Soil | DRZ |
| Shallow bulk soil | Soil | SBS |
| Deep bulk soil | Soil | DBS |

**Table S2.** Primer mixtures and PNA PCR blockers used in this study. Sequences in blue represent Nextera annealing sites, black represents the Molecular Identifier Tag including frameshifts, green represents linker adaptors, and red represents PCR primers.

| **PRIMER NAME** | **SEQUENCE** | **DIRECTION** | **TARGET** | **REFERENCE** |
| --- | --- | --- | --- | --- |
| ITS3NGS1-F1 | TCCCTCGCGCCATCAGAGATGTG TATAAGAGACAG NNNNNNNN TT CATCGATGAAGAACGCAG | Forward | Fungi | White et al. 1990 |
| ITS3NGS1-F2 | TCCCTCGCGCCATCAGAGATGTG TATAAGAGACAG NNNNTNNNN TT CATCGATGAAGAACGCAG | Forward | Fungi | White et al. 1990 |
| ITS3NGS1-F3 | TCCCTCGCGCCATCAGAGATGTG TATAAGAGACAG NNNNCTNNNN TT CATCGATGAAGAACGCAG | Forward | Fungi | White et al. 1990 |
| ITS3NGS1-F4 | TCCCTCGCGCCATCAGAGATGTG TATAAGAGACAG NNNNACTNNNN TT CATCGATGAAGAACGCAG | Forward | Fungi | White et al. 1990 |
| ITS3NGS1-F5 | TCCCTCGCGCCATCAGAGATGTG TATAAGAGACAG NNNNGACTNNNN TT CATCGATGAAGAACGCAG | Forward | Fungi | White et al. 1990 |
| ITS3NGS1-F6 | TCCCTCGCGCCATCAGAGATGTG TATAAGAGACAG NNNNTGACTNNNN TT CATCGATGAAGAACGCAG | Forward | Fungi | White et al. 1990 |
| ITS3NGS2-F1 | TCCCTCGCGCCATCAGAGATGTG TATAAGAGACAG NNNNNNNN TT CAACGATGAAGAACGCAG | Forward | Chytridiomycota | Tedersoo et al. 2014 |
| ITS3NGS3-F2 | TCCCTCGCGCCATCAGAGATGTG TATAAGAGACAG NNNNTNNNN TT CACCGATGAAGAACGCAG | Forward | Sebacinales | Tedersoo et al. 2014 |
| ITS3NGS4-F3 | TCCCTCGCGCCATCAGAGATGTG TATAAGAGACAG NNNNCTNNNN TT CATCGATGAAGAACGTAG | Forward | Glomeromycota | Tedersoo et al. 2014 |
| ITS3NGS5-F4 | TCCCTCGCGCCATCAGAGATGTG TATAAGAGACAG NNNNACTNNNN TT CATCGATGAAGAACGTGG | Forward | Sordariales | Tedersoo et al. 2014 |
| ITS3NGS10-F5 | TCCCTCGCGCCATCAGAGATGTG TATAAGAGACAG NNNNGACTNNNN TT CATCGATGAAGAACGCTG | Forward | Stramenopila | Tedersoo et al. 2014 |
| ITS4NGR-F1 | GTGACTGGAGTTCAGACGTGTGCTC TTCCGATCT NNNNN GA TCCTSCGCTTATTGATATGC | Reverse | Fungi | White et al. 1990 |
| ITS4NGR-F2 | GTGACTGGAGTTCAGACGTGTGCTC TTCCGATCT NNTNNN GA TCCTSCGCTTATTGATATGC | Reverse | Fungi | White et al. 1990 |
| ITS4NGR-F3 | GTGACTGGAGTTCAGACGTGTGCTC TTCCGATCT NNCTNNN GA TCCTSCGCTTATTGATATGC | Reverse | Fungi | White et al. 1990 |
| ITS4NGR-F4 | GTGACTGGAGTTCAGACGTGTGCTC TTCCGATCT NNACTNNN GA TCCTSCGCTTATTGATATGC | Reverse | Fungi | White et al. 1990 |
| ITS4NGR-F5 | GTGACTGGAGTTCAGACGTGTGCTC TTCCGATCT NNGACTNNN GA TCCTSCGCTTATTGATATGC | Reverse | Fungi | White et al. 1990 |
| ITS4NGR-F6 | GTGACTGGAGTTCAGACGTGTGCTC TTCCGATCT NNTGACTNNN GA TCCTSCGCTTATTGATATGC | Reverse | Fungi | White et al. 1990 |
| ARCH-ITS4-F1 | GTGACTGGAGTTCAGACGTGTGCTC TTCCGATCT NNNNN GA TCCTCGCCTTATTGATATGC | Reverse | Archaearhizomycetes | This paper |
| 515F_f1 | GCCTCCCTCGCGCCATCAGAGATGTGTATAAGAGACAGNNNNNNNNGAGTGCCAGCMGCCGCGGTAA | Forward | Bacteria/Archaea | Lane et al. 1985 |
| 515F_f2 | GCCTCCCTCGCGCCATCAGAGATGTGTATAAGAGACAGNNNNTNNNNGAGTGCCAGCMGCCGCGGTAA | Forward | Bacteria/Archaea | Lane et al. 1985 |
| 515F_f3 | GCCTCCCTCGCGCCATCAGAGATGTGTATAAGAGACAGNNNNCTNNNNGAGTGCCAGCMGCCGCGGTAA | Forward | Bacteria/Archaea | Lane et al. 1985 |
| 515F_f4 | GCCTCCCTCGCGCCATCAGAGATGTGTATAAGAGACAGNNNNACTNNNNGAGTGCCAGCMGCCGCGGTAA | Forward | Bacteria/Archaea | Lane et al. 1985 |
| 515F_f5 | GCCTCCCTCGCGCCATCAGAGATGTGTATAAGAGACAGNNNNGACTNNNNGAGTGCCAGCMGCCGCGGTAA | Forward | Bacteria/Archaea | Lane et al. 1985 |
| 515F_f6 | GCCTCCCTCGCGCCATCAGAGATGTGTATAAGAGACAGNNNNTGACTNNNNGAGTGCCAGCMGCCGCGGTAA | Forward | Bacteria/Archaea | Lane et al. 1985 |
| 515F_f1C | GCCTCCCTCGCGCCATCAGAGATGTGTATAAGAGACAGNNNNNNNNGAGTGCCAGCMGCWGCGGTAA | Forward | Cloroflexi | Shakya et al. 2013 |
| 515F_f1TM7 | GCCTCCCTCGCGCCATCAGAGATGTGTATAAGAGACAGNNNNNNNNGAGTGCCAGCMGCCGCGGTCA | Forward | TM7 | Shakya et al. 2013 |
| 515F_f4Arc | GCCTCCCTCGCGCCATCAGAGATGTGTATAAGAGACAGNNNNACTNNNNGAGTGKCAGCMGCCGCGGTAA | Forward | Archaea | Shakya et al. 2013 |
| 806R_f1 | GTGACTGGAGTTCAGACGTGTGCTCTTCCGATCTNNNNNACGGACTACHVGGGTWTCTAAT | Reverse | Bacteria/Archaea | Lane et al. 1985 |
| 806R_f2 | GTGACTGGAGTTCAGACGTGTGCTCTTCCGATCTNNTNNNACGGACTACHVGGGTWTCTAAT | Reverse | Bacteria/Archaea | Lane et al. 1985 |
| 806R_f3 | GTGACTGGAGTTCAGACGTGTGCTCTTCCGATCTNNCTNNNACGGACTACHVGGGTWTCTAAT | Reverse | Bacteria/Archaea | Lane et al. 1985 |
| 806R_f4 | GTGACTGGAGTTCAGACGTGTGCTCTTCCGATCTNNACTNNNACGGACTACHVGGGTWTCTAAT | Reverse | Bacteria/Archaea | Lane et al. 1985 |
| 806R_f5 | GTGACTGGAGTTCAGACGTGTGCTCTTCCGATCTNNGACTNNNACGGACTACHVGGGTWTCTAAT | Reverse | Bacteria/Archaea | Lane et al. 1985 |
| 806R_f6 | GTGACTGGAGTTCAGACGTGTGCTCTTCCGATCTNNTGACTNNNACGGACTACHVGGGTWTCTAAT | Reverse | Bacteria/Archaea | Lane et al. 1985 |
| pPNA | GGCTCAACCCTGGACAG | NA | Plants (Plastid) | Lundberg et al. 2013 |
| Pop-mPNA | GGCAAGTCTTCTTCGGA | NA | Populus (Mitochondrial) | Lundberg et al. 2013 (modified) |
| Plant-ITS-PNA | CGAGGGCACGTCTGCCTGG | NA | Dicots (Nuclear) | This paper |

**Table S3.** Two-way ANOVA (habitat X genotype) p values for Tukey’s HSD post-hoc pairwise comparisons test in *Septoria sp.* relative abundance differences across leaf habitats. Models indicate that *Septoria sp.* differed across habitats (F = 9.34, p = < 0.01) and tree genotypes (F = 56.34, p = < 0.01).

|  | **LPD** | **DWL** | **LED** | **PED** | **UPM** | **LPM** | **MWL** | **LEM** | **PEM** |
| --- | --- | --- | --- | --- | --- | --- | --- | --- | --- |
| **LPD** |  | 0.98 | 1.00 | 0.74 | 0.43 | 0.84 | 0.07 | **<0.01** | 1.00 |
| **DWL** | 0.98 |  | 0.84 | 1.00 | **0.04** | 0.21 | **<0.01** | **<0.01** | 1.00 |
| **LED** | 1.00 | 0.84 |  | 0.45 | 0.79 | 0.99 | 0.26 | **<0.01** | 0.99 |
| **PED** | 0.74 | 1.00 | 0.45 |  | **<0.01** | **0.04** | **<0.01** | **<0.01** | 0.95 |
| **UPM** | 0.43 | **0.04** | 0.79 | **<0.01** |  | 1.00 | 0.99 | **0.03** | 0.17 |
| **LPM** | 0.84 | 0.21 | 0.99 | **0.04** | 1.00 |  | 0.85 | **<0.01** | 0.53 |
| **MWL** | 0.07 | **<0.01** | 0.26 | **<0.01** | 0.99 | 0.85 |  | 0.22 | **0.02** |
| **LEM** | **<0.01** | **<0.01** | **<0.01** | **<0.01** | **0.03** | **<0.01** | 0.22 |  | **<0.01** |
| **PEM** | 1.00 | 1.00 | 0.99 | 0.95 | 0.17 | 0.53 | **0.02** | **<0.01** |  |
|  |  |  |  |  |  |  |  |  |  |

**Table S4.** Two-way ANOVA (habitat X genotype) p values for Tukey’s HSD post-hoc pairwise comparisons test in *Marssonina brunnea.* relative abundance differences across leaf habitats. Models indicate that *Marssonina.* differed across habitats (F = 6.40, p = < 0.01) and tree genotypes (F = 590.95, p = < 0.01).

|  | **LPD** | **DWL** | **LED** | **PED** | **UPM** | **LPM** | **MWL** | **LEM** | **PEM** |
| --- | --- | --- | --- | --- | --- | --- | --- | --- | --- |
| **LPD** |  | 0.36 | **<0.01** | 0.06 | 1.00 | 0.56 | 0.40 | **<0.01** | 0.93 |
| **DWL** | 0.36 |  | 0.49 | 0.99 | 0.67 | 1.00 | 1.00 | **0.02** | 0.98 |
| **LED** | **<0.01** | 0.49 |  | 0.94 | **<0.01** | 0.30 | 0.45 | 0.78 | 0.07 |
| **PED** | 0.06 | 0.99 | 0.94 |  | 0.18 | 0.96 | 0.99 | 0.12 | 0.65 |
| **UPM** | 1.00 | 0.67 | **<0.01** | 0.18 |  | 0.85 | 0.71 | **<0.01** | 1.00 |
| **LPM** | 0.56 | 1.00 | 0.30 | 0.96 | 0.85 |  | 1.00 | **<0.01** | 1.00 |
| **MWL** | 0.40 | 1.00 | 0.45 | 0.99 | 0.71 | 1.00 |  | **0.01** | 1.00 |
| **LEM** | **<0.01** | **0.02** | 0.78 | 0.12 | **<0.01** | **<0.01** | **0.01** |  | **<0.01** |
| **PEM** | 0.93 | 0.98 | 0.07 | 0.65 | 1.00 | 1.00 | 1.00 | **<0.01** |  |
|  |  |  |  |  |  |  |  |  |  |

**Table S5.** Two-way ANOVA (habitat X genotype) p values for pairwise comparisons in bacterial diversity across leaf niches. Two-way ANOVA models indicate that bacterial diversity differed within habitats (F = 2.53, p = 0.013), but not between tree genotypes (F = 0.003, p = 0.958).

|  | **UPD** | **LPD** | **DWL** | **LED** | **PED** | **UPM** | **LPM** | **MWL** | **LEM** | **PEM** |
| --- | --- | --- | --- | --- | --- | --- | --- | --- | --- | --- |
| **UPD** |  | 0.98 | **0.02** | 1.00 | 0.38 | 1.00 | 0.97 | 0.85 | 1.00 | 1.00 |
| **LPD** | 0.98 |  | 0.28 | 1.00 | 0.94 | 1.00 | 1.00 | 1.00 | 0.98 | 1.00 |
| **DWL** | **0.02** | 0.28 |  | 0.22 | 1.00 | 0.11 | 0.33 | 0.57 | **0.02** | **0.05** |
| **LED** | 1.00 | 1.00 | 0.22 |  | 0.86 | 1.00 | 1.00 | 1.00 | 1.00 | 1.00 |
| **PED** | 0.38 | 0.94 | 1.00 | 0.86 |  | 0.78 | 0.96 | 0.99 | 0.38 | 0.60 |
| **UPM** | 1.00 | 1.00 | 0.11 | 1.00 | 0.78 |  | 1.00 | 1.00 | 1.00 | 1.00 |
| **LPM** | 0.97 | 1.00 | 0.33 | 1.00 | 0.96 | 1.00 |  | 1.00 | 0.96 | 1.00 |
| **MWL** | 0.85 | 1.00 | 0.57 | 1.00 | 0.99 | 1.00 | 1.00 |  | 0.84 | 0.97 |
| **LEM** | 1.00 | 0.98 | **0.02** | 1.00 | 0.38 | 1.00 | 0.96 | 0.84 |  | 1.00 |
| **PEM** | 1.00 | 1.00 | **0.05** | 1.00 | 0.60 | 1.00 | 1.00 | 0.97 | 1.00 |  |

**Table S6:** Two-way ANOVA (habitat X genotype) p values for pairwise comparisons in bacterial diversity across stem niches. Two-way ANOVA models indicate that bacterial diversity differed within habitats (F = 2.984, p = 0.006), but not between tree genotypes (F = 1.386, p = 0.243).

|  | **1HX** | **1DX** | **1CB** | **2HX** | **2DX** | **2CB** | **3HX** | **3DX** | **3CB** |
| --- | --- | --- | --- | --- | --- | --- | --- | --- | --- |
| **1HX** |  | **0.02** | **0.04** | 0.09 | 0.25 | 0.30 | 0.33 | 1.00 | 0.93 |
| **1DX** | **0.02** |  | 1.00 | 1.00 | 0.98 | 0.97 | 0.99 | 0.10 | 0.38 |
| **1CB** | **0.04** | 1.00 |  |  | 1.00 | 0.99 | 1.00 | 0.57 | 0.57 |
| **2HX** | 0.09 | 1.00 |  |  | 1.00 | 1.00 | 1.00 | 0.31 | 0.72 |
| **2DX** | 0.25 | 0.98 | 1.00 | 1.00 |  | 1.00 | 1.00 | 0.62 | 0.95 |
| **2CB** | 0.30 | 0.97 | 0.99 | 1.00 | 1.00 |  | 1.00 | 0.68 | 0.97 |
| **3HX** | 0.33 | 0.99 | 1.00 | 1.00 | 1.00 | 1.00 |  | 0.69 | 0.97 |
| **3DX** | 1.00 | 0.10 | 0.18 | 0.31 | 0.62 | 0.68 | 0.69 |  | 1.00 |
| **3CB** | 0.93 | 0.38 | 0.57 | 0.72 | 0.95 | 0.97 | 0.97 | 1.00 |  |

**Table S7:** Two-way ANOVA (habitat X genotype) p values for pairwise comparisons in bacterial diversity across root niches. Two-way ANOVA models indicate that bacterial diversity differed within habitats (F = 11.474, p < 0.001), but not between tree genotypes (F = 0.987, p = 0.324).

|  | **SFR** | **DFR** | **SSR** | **DSR** | **SHX** | **SDX** | **SCB** |
| --- | --- | --- | --- | --- | --- | --- | --- |
| **SFR** |  | 1.00 | 0.98 | 0.99 | **<0.01** | **<0.01** | 0.95 |
| **DFR** | 1.00 |  | 0.99 | 1.00 | **<0.01** | **<0.01** | 0.98 |
| **SSR** | 0.98 | 0.99 |  | 1.00 | **<0.01** | **<0.01** | 1.00 |
| **DSR** | 0.99 | 1.00 | 1.00 |  | **<0.01** | **<0.01** | 1.00 |
| **SHX** | **<0.01** | **<0.01** | **<0.01** | **<0.01** |  | 0.98 | **<0.01** |
| **SDX** | **<0.01** | **<0.01** | **<0.01** | **<0.01** | 0.98 |  | **<0.01** |
| **SCB** | 0.95 | 0.98 | 1.00 | 1.00 | **<0.01** | **<0.01** |  |

**Table S8:** Two-way ANOVA (habitat X genotype) p values for pairwise comparisons in bacterial diversity across soil niches. Two-way ANOVA models indicate that bacterial diversity differed within habitats (F = 7.821, p < 0.001), but not between tree genotypes (F = 0.297, p = 0.589).

|  | **SRZ** | **DRZ** | **SBS** | **DBS** |
| --- | --- | --- | --- | --- |
| **SRZ** |  | 0.94 | **0.01** | **0.04** |
| **DRZ** | 0.94 |  | **<0.01** | **0.01** |
| **SBS** | **0.01** | **<0.01** |  | 0.90 |
| **DBS** | **0.04** | **0.01** | 0.90 |  |

**Table S9:** Two-way ANOVA (habitat X genotype) p values for pairwise comparisons in fungal diversity across leaf niches. Two-way ANOVA models indicate that fungal diversity differed within habitats (F = 8.198, p < 0.001), and between tree genotypes (F = 86.509, p < 0.001).

|  | **UPD** | **LPD** | **DWL** | **LED** | **PED** | **UPM** | **LPM** | **MWL** | **LEM** | **PEM** |
| --- | --- | --- | --- | --- | --- | --- | --- | --- | --- | --- |
| **UPD** |  |  |  |  |  |  |  |  |  |  |
| **LPD** |  |  | 0.91 | **<0.01** | 0.06 | 0.99 | 0.88 | 0.96 | **<0.01** | 0.49 |
| **DWL** |  | 0.91 |  | **<0.01** | 0.71 | 0.36 | 1.00 | 1.00 | 0.16 | 1.00 |
| **LED** |  | **<0.01** | **<0.01** |  | 0.17 | **<0.01** | **<0.01** | **<0.01** | 0.91 | 0.01 |
| **PED** |  | 0.06 | 0.71 | 0.17 |  | **<0.01** | 0.74 | 0.58 | 0.97 | 0.98 |
| **UPM** |  | 0.99 | 0.36 | **<0.01** | **<0.01** |  | 0.32 | 0.48 | **<0.01** | 0.08 |
| **LPM** |  | 0.88 | 1.00 | **<0.01** | 0.74 | 0.32 |  | 1.00 | 0.18 | 1.00 |
| **MWL** |  | 0.96 | 1.00 | **<0.01** | 0.58 | 0.48 | 1.00 |  | 0.10 | 0.99 |
| **LEM** |  | **<0.01** | 0.16 | 0.91 | 0.97 | **<0.01** | 0.18 | 0.18 |  | 0.50 |
| **PEM** |  | 0.49 | 1.00 | 0.01 | 0.98 | 0.08 | 1.00 | 0.99 | 0.50 |  |

**Table S10:** Two-way ANOVA (habitat X genotype) p values for pairwise comparisons in fungal diversity across stem niches. Two-way ANOVA models indicate that fungal diversity differed within habitats (F = 4.568, p < 0.001), and between tree genotypes (F = 6.127, p = 0.015).

|  | **1HX** | **1DX** | **1CB** | **2HX** | **2DX** | **2CB** | **3HX** | **3DX** | **3CB** |
| --- | --- | --- | --- | --- | --- | --- | --- | --- | --- |
| **1HX** |  | 0.93 | **0.01** | 1.00 | 1.00 | 0.31 | 1.00 | 1.00 | 1.00 |
| **1DX** | 0.93 |  | 0.22 | 0.76 | 0.53 | 0.97 | 0.98 | 0.87 | 0.94 |
| **1CB** | **0.01** |  |  | **<0.01** | **<0.01** | 0.87 | **0.02** | **<0.01** | **0.01** |
| **2HX** | 1.00 | 0.76 | **<0.01** |  | 1.00 | 0.14 | 1.00 | 1.00 | 1.00 |
| **2DX** | 1.00 | 0.53 | **<0.01** | 1.00 |  | 0.06 | 1.00 | 1.00 | 1.00 |
| **2CB** | 0.31 | 0.97 | 0.87 | 0.14 | 0.06 |  | 0.48 | 0.22 | 0.32 |
| **3HX** | 1.00 | 0.98 | **0.02** | 1.00 | 1.00 | 0.48 |  | 1.00 | 1.00 |
| **3DX** | 1.00 | 0.87 | **<0.01** | 1.00 | 1.00 | 0.22 | 1.00 |  | 1.00 |
| **3CB** | 1.00 | 0.94 | **0.01** | 1.00 | 1.00 | 0.32 | 1.00 | 1.00 |  |

**Table S11:** Two-way ANOVA (habitat X genotype) p values for pairwise comparisons in fungal diversity across soil niches. Two-way ANOVA models indicate that fungal diversity differed within habitats (F = 6.026, p = 0.002), but not between tree genotypes (F = 0.036, p = 0.851).

|  | **SRZ** | **DRZ** | **SBS** | **DBS** |
| --- | --- | --- | --- | --- |
| **SRZ** |  | 0.15 | 0.81 | **<0.01** |
| **DRZ** | 0.15 |  | 0.56 | 0.17 |
| **SBS** | 0.81 | 0.56 |  | **0.01** |
| **DBS** | **<0.01** | 0.17 | **0.01** |  |

**Table S12.** Relative abundance of dominant (≥0.1%) archaeal/bacterial and fungal phyla, and class for Proteobacteria across broad habitat categories and genotypes (mean ± SE). Two-way ANOVA models indicated all bacterial and fungal phyla, except Fusobacteria, differed across habitat (p≤0.01) whereas two bacterial phyla differed between genotypes (p≤0.03) as denoted by bolded lettering. Letters denotes Tukey’s HSD significant differences for main effects of habitat and genotype.

|  | ***Habitat*** | | | | ***Genotype*** | |
| --- | --- | --- | --- | --- | --- | --- |
| **Phylum** | **Leaves** | **Stems** | **Roots** | **Soil** | **DD** | **TD** |
| *Bacteria* |  |  |  |  |  |  |
| Crenarchaeota | 0.22 (0.07)^a^ | 0.28 (0.13)^a^ | 0.26 (0.06)^a^ | 3.02 (0.33)^b^ | 0.72 (0.13) | 0.61 (0.12) |
| Alphaproteobacteria | 42.35 (3.08)^a^ | 57.91 (2.19)^b^ | 18.94 (1.69)^c^ | 9.10 (0.51)^d^ | 34.40 (2.36) | 37.56 (2.31) |
| Betaproteobacteria | 3.19 (0.59)^a^ | 0.92 (0.11)^b^ | 4.99 (0.60)^c^ | 6.99 (0.32)^d^ | 3.82 (0.39) | 3.29 (0.38) |
| Deltaproteobacteria | 1.17 (0.15)^a^ | 0.47 (0.11)^a^ | 4.03 (0.52)^b^ | 6.04 (0.41)^c^ | 2.51 (0.29) | 2.32 (0.27) |
| Gammaproteobacteria | 41.67 (3.32)^a^ | 9.50 (2.21)^b^ | 31.26 (3.73)^a^ | 15.13 (2.53)^b^ | 25.0 (2.45) | 26.51 (2.56) |
| Acidobacteria | 0.63 (0.16)^a^ | 0.32 (0.15)^a^ | 1.97 (0.16)^b^ | 14.20 (1.18)^c^ | 2.81 (0.46) | 2.99 (0.52) |
| **Actinobacteria** | 4.95 (0.73)^a^ | 19.52 (0.95)^b^ | 23.48 (1.96)^b^ | 15.17 (0.90)^b^ | 17.70 (1.16)^a^ | 13.10 (0.97)^b^ |
| AD3 | 0.06 (0.02)^a^ | 0.02 (0.01)^a^ | 0.007 (0.004)^a^ | 3.16 (0.71)^b^ | 0.37 (0.14) | 0.62 (0.20) |
| Armatimonadetes | 0.07 (0.02)^a^ | 0.32 (0.05)^b^ | 0.25 (0.04)^b^ | 0.35 (0.04)^b^ | 0.22 (0.02) | 0.25 (0.03) |
| Bacteroidetes | 3.04 (0.40)^a^ | 7.47 (0.62)^b^ | 7.39 (0.73)^b^ | 2.78 (0.31)^a^ | 4.96 (0.34) | 5.81 (0.54) |
| Chloroflexi | 0.23 (0.05)^a^ | 0.19 (0.05)^a^ | 1.89 (0.37)^b^ | 4.43 (0.26)^b^ | 1.27 (0.15) | 1.25 (0.23) |
| FBP | 0.008 (0.004)^a^ | 0.46 (0.07)^b^ | 0.02 (0.007)^a^ | 0.02 (0.008)^a^ | 0.17 (0.04) | 0.12 (0.02) |
| Firmicutes | 0.67 (0.08)^a^ | 1.33 (0.62)^a^ | 0.80 (0.16)^a^ | 2.28 (0.24)^b^ | 1.52 (0.38) | 0.76 (0.09) |
| Fusobacteria | 0.04 (0.02) | 0.32 (0.21) | 0.006 (0.004) | 0 | 0.20 (0.12) | 0.02 (0.01) |
| Gemmatimonadetes | 0.13 (0.03)^ab^ | 0.02 (0.02)^a^ | 0.18 (0.02)^b^ | 3.41 (0.33)^c^ | 0.67 (0.14) | 0.53 (0.11) |
| Nitrospirae | 0.12 (0.014)^a^ | 0.06 (0.04)^a^ | 0.10 (0.02)^a^ | 5.19 (0.62)^b^ | 0.86 (0.21) | 0.84 (0.20 |
| Planctomycetes | 0.17 (0.03)^a^ | 0.12 (0.03)^a^ | 1.48 (0.14)^b^ | 2.91 (0.21)^b^ | 0.98 (0.12) | 0.81 (0.10) |
| TM6 | 0.42 (0.10)^a^ | 0.02 (0.007)^b^ | 0.21 (0.04)^a^ | 0.20 (0.03)^a^ | 0.20 (0.04) | 0.24 (0.06) |
| **TM7** | 0.10 (0.03)^a^ | 0.12 (0.03)^a^ | 0.84 (0.16)^b^ | 0.14 (0.03)^a^ | 0.17 (0.03)^a^ | 0.43 (0.09)^b^ |
| Verrucomicrobia | 0.20 (0.05)^a^ | 0.15 (0.03)^a^ | 1.36 (0.13)^b^ | 2.67 (0.19)^c^ | 0.75 (0.09) | 0.94 (0.11) |
| WS3 | 0.05 (0.02)^a^ | 0.01 (0.006)^a^ | 0.03 (0.008)^a^ | 1.45 (0.13)^b^ | 0.27 (0.06) | 0.22 (0.05) |
| *Fungi* |  |  |  |  |  |  |
| Ascomycota | 83.83 (1.42)^a^ | 68.46 (1.32)^b^ | 71.03 (2.55)^b^ | 60.0 (2.31)^c^ | 71.14 (1.29) | 73.75 (1.59) |
| Basidiomycota | 8.43 (0.89)^a^ | 21.35 (1.26)^b^ | 12.39 (1.84)^a^ | 6.47 (0.99)^a^ | 13.67 (0.98) | 13.65 (1.12) |
| Chytridiomycota | 0.03 (0.008)^a^ | 3.65 (0.57)^b^ | 0.53 (0.11)^a^ | 1.11 (0.13)^a^ | 1.35 (0.31) | 1.53 (0.27) |
| Glomeromycota | 0.03 (0.008)^a^ | 3.59 (0.56)^b^ | 0.53 (0.10)^a^ | 0.93 (0.18)^a^ | 1.30 (0.24) | 1.49 (0.32) |
| Rozellomycota | 0.006 (0.004)^a^ | 0.002 (0.001)^a^ | 0.04 (0.01)^a^ | 0.93 (0.39)^b^ | 0.03 (0.006) | 0.26 (0.12) |
| Zygomycota | 0.11 (0.04)^a^ | 0.22 (0.07)^a^ | 11.48 (1.98)^b^ | 26.76 (2.64)^c^ | 7.17 (1.18) | 6.29 (1.17) |

**Figure S1.** Sampling schema for 30 plant niches. Each niche was sampled from five replicate *Populus deltoides* clones and *P. trichocarpa x deltoides* hybrid clones, totaling 300 microbiome samples.


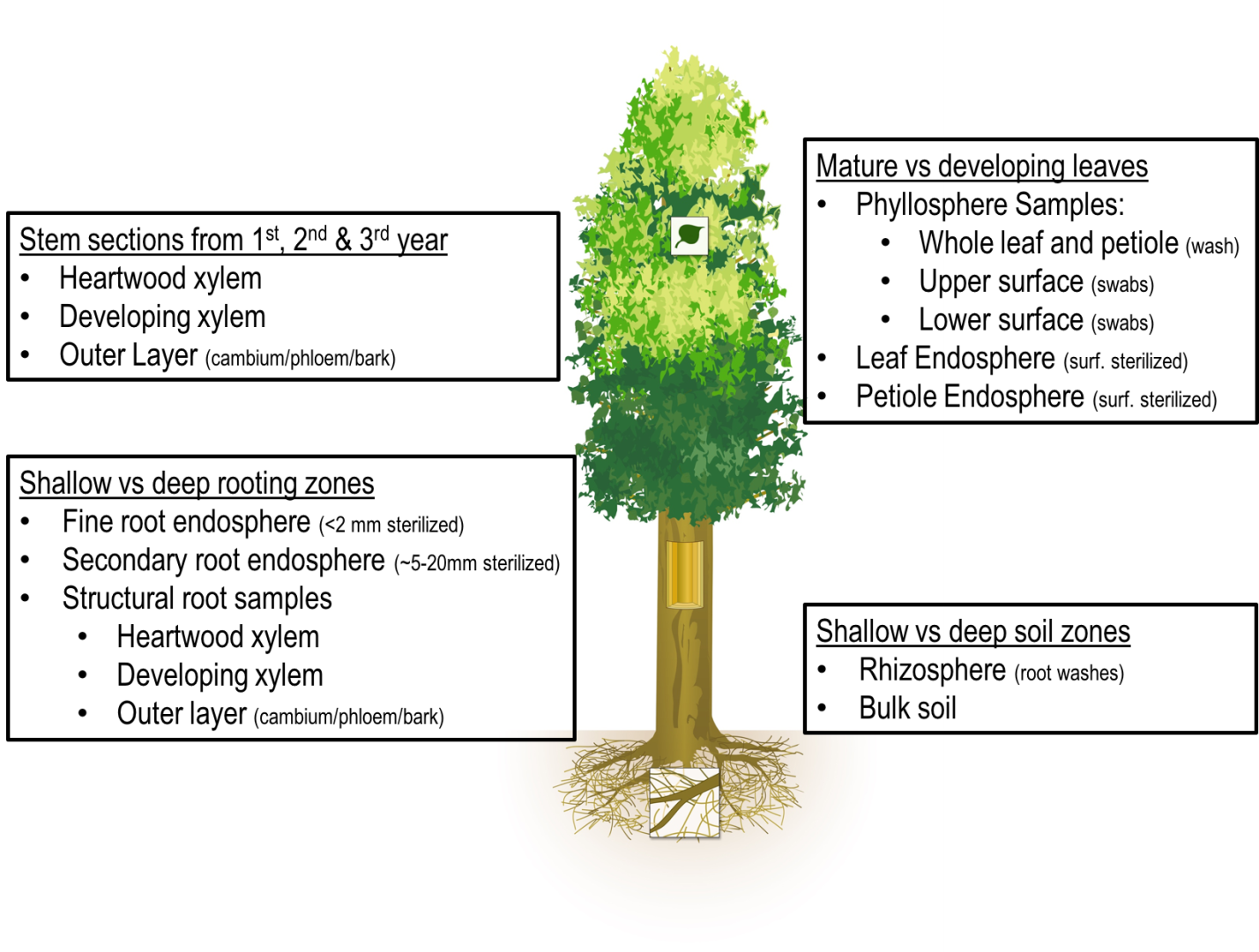


**Figure S2.** Performance of plant nuclear 5.8S rRNA gene targeted peptide nucleic acid (PNA) blocker in select fungal ITS2 amplicon libraries. We used two different tissue types including fine root endosphere (SFR), developing leaf endosphere (LED), as well as rhizosphere soils. These are tested on samples originating from *Populus deltoides* (DD1) and a *P. trichocarpa X deltoides* hybrid (TD1). Samples with PNA blockers added indicated by _PNA at end of name.

**Figure S3.** Rarefaction curves for bacteria across broad habitat classifications (leaves – red, stems – green, roots – blue, soil – orange) at 1000 sequences per sample depth.


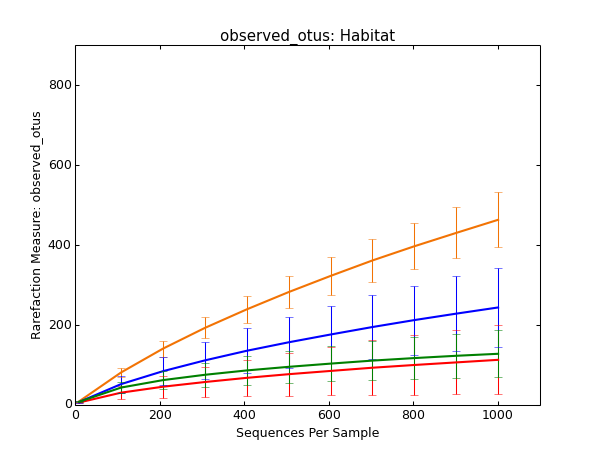


**Figure S4.** Rarefaction curves for fungi across broad habitat classifications (leaves – red, stems – green, roots – blue, soil – orange) at 2000 sequences per sample depth.


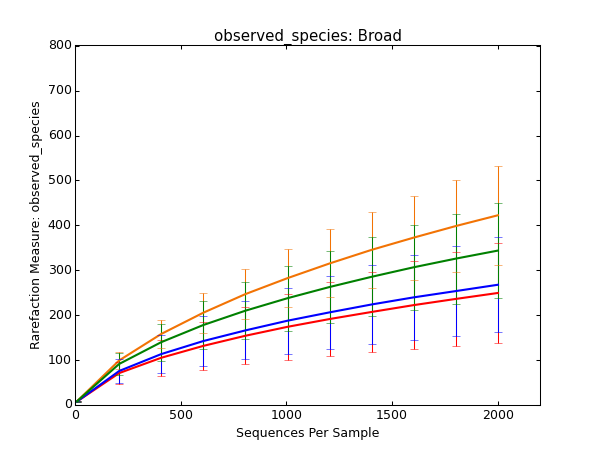


Bacterial Data Processing:

##Load parameters

source orion.bashrc

##Join each runs reads

join_paired_ends.py -b /illumina/MiSeqDawn/MiSeqAnalysis/150417_Schadt_16s_Atlas_1_AB977/Data/Intensities/BaseCalls/Undetermined_S0_L001_I1_001.fastq -f /illumina/MiSeqDawn/MiSeqAnalysis/150417_Schadt_16s_Atlas_1_AB977/Data/Intensities/BaseCalls/Undetermined_S0_L001_R1_001.fastq -r /illumina/MiSeqDawn/MiSeqAnalysis/150417_Schadt_16s_Atlas_1_AB977/Data/Intensities/BaseCalls/Undetermined_S0_L001_R2_001.fastq -o Run1joined_reads/

join_paired_ends.py -b /illumina/MiSeqDawn/MiSeqAnalysis/150610_UT_DD1_16s-AF69G/Data/Intensities/BaseCalls/Undetermined_S0_L001_I1_001.fastq.gz -f /illumina/MiSeqDawn/MiSeqAnalysis/150610_UT_DD1_16s-AF69G/Data/Intensities/BaseCalls/Undetermined_S0_L001_R1_001.fastq.gz -r /illumina/MiSeqDawn/MiSeqAnalysis/150610_UT_DD1_16s-AF69G/Data/Intensities/BaseCalls/Undetermined_S0_L001_R2_001.fastq.gz -o Run2joined_reads/

join_paired_ends.py -b /illumina/MiSeqDawn/MiSeqAnalysis/150713_M02014_Melissa-Zamin_AF6NA/Data/Intensities/BaseCalls/Undetermined_S0_L001_I1_001.fastq.gz -f /illumina/MiSeqDawn/MiSeqAnalysis/150713_M02014_Melissa-Zamin_AF6NA/Data/Intensities/BaseCalls/Undetermined_S0_L001_R1_001.fastq.gz -r /illumina/MiSeqDawn/MiSeqAnalysis/150713_M02014_Melissa-Zamin_AF6NA/Data/Intensities/BaseCalls/Undetermined_S0_L001_R2_001.fastq.gz -o Run3joined_reads/

join_paired_ends.py -b /illumina/MiSeqDawn/MiSeqAnalysis/150810_Melissa-Zamin_16s_AF69L/Data/Intensities/BaseCalls/Undetermined_S0_L001_I1_001.fastq.gz -f /illumina/MiSeqDawn/MiSeqAnalysis/150810_Melissa-Zamin_16s_AF69L/Data/Intensities/BaseCalls/Undetermined_S0_L001_R1_001.fastq.gz -r /illumina/MiSeqDawn/MiSeqAnalysis/150810_Melissa-Zamin_16s_AF69L/Data/Intensities/BaseCalls/Undetermined_S0_L001_R2_001.fastq.gz  -o Run4joined_reads/

join_paired_ends.py -b /illumina/MiSeqDawn/MiSeqAnalysis/151029_PMI-bacterial_Rerun_AJPF7/Data/Intensities/BaseCalls/Undetermined_S0_L001_I1_001.fastq.gz -f /illumina/MiSeqDawn/MiSeqAnalysis/151029_PMI-bacterial_Rerun_AJPF7/Data/Intensities/BaseCalls/Undetermined_S0_L001_R1_001.fastq.gz -r /illumina/MiSeqDawn/MiSeqAnalysis/151029_PMI-bacterial_Rerun_AJPF7/Data/Intensities/BaseCalls/Undetermined_S0_L001_R2_001.fastq.gz -o Run5joined_reads/

##Split libraries

split_libraries_fastq.py -i Run1joined_reads/fastqjoin.join.fastq -b Run1joined_reads/fastqjoin.join_barcodes.fastq -m PMIAtlasMappingRun1.txt --store_demultiplexed_fastq --rev_comp_mapping_barcodes -q 19 -o Run1joined_reads/sl_out

split_libraries_fastq.py -i Run2joined_reads/fastqjoin.join.fastq -b Run2joined_reads/fastqjoin.join_barcodes.fastq -m PMIAtlasMappingRun2.txt --store_demultiplexed_fastq --rev_comp_mapping_barcodes -q 19 -o Run2joined_reads/sl_out

##Concatenate together using cat command

cutadapt -n 2 -g GAGTGCCAGCMGCCGCGGTAA -g GAGTGCCAGCMGCWGCGGTAA -g GAGTGCCAGCMGCCGCGGTCA -g GAGTGKCAGCMGCCGCGGTAA -g GAGTGGCAGYCGCCRCGGKAA -a ATTAGAWACCCBDGTAGTCCGT -a ATTAGAGACCCCDGTAKTCCGT -e 0.1 --discard-untrimmed --match-read-wildcards -o Trimmed_Rev.fastq Combined_seqs.fastq

This is cutadapt 1.9.dev0 with Python 2.7.6

Command line parameters: -n 2 -g GAGTGCCAGCMGCCGCGGTAA -g GAGTGCCAGCMGCWGCGGTAA -g GAGTGCCAGCMGCCGCGGTCA -g GAGTGKCAGCMGCCGCGGTAA -g GAGTGGCAGYCGCCRCGGKAA -a ATTAGAWACCCBDGTAGTCCGT -a ATTAGAGACCCCDGTAKTCCGT -e 0.1 --discard-untrimmed --match-read-wildcards -o Trimmed_Rev.fastq Combined_seqs.fastq

Trimming 7 adapter(s) with at most 10.0% errors in single-end mode ...

Finished in 20940.33 s (411 us/read; 0.15 M reads/minute).

=== Summary ===

Total reads processed:              50,996,814

Reads with adapters:               101,876,660 (199.8%)

Reads written (passing filters):    50,995,823 (100.0%)

Total basepairs processed: 16,025,524,935 bp

Total written (filtered):  12,918,265,869 bp (80.6%)

=== Adapter 1 ===

Sequence: ATTAGAWACCCBDGTAGTCCGT; Type: regular 3'; Length: 22; Trimmed: 50906522 times.

No. of allowed errors:

0-9 bp: 0; 10-19 bp: 1; 20-22 bp: 2

Bases preceding removed adapters:

  A: 0.0%

  C: 0.0%

  G: 99.9%

  T: 0.1%

  none/other: 0.0%

WARNING:

    The adapter is preceded by "G" extremely often.

    The provided adapter sequence may be incomplete.

    To fix the problem, add "G" to the beginning of the adapter sequence.

Overview of removed sequences

length count expect max.err error counts

3 1346 796825.2 0 1346

4 111 199206.3 0 111

5 34 49801.6 0 34

6 5 12450.4 0 5

7 12 3112.6 0 12

8 2 778.1 0 2

9 4 194.5 0 4

10 25 48.6 1 15 10

11 37 12.2 1 29 8

12 80 3.0 1 75 5

13 75 0.8 1 52 23

14 109 0.2 1 49 60

15 792 0.0 1 705 87

16 254 0.0 1 151 103

17 323 0.0 1 186 137

18 542 0.0 1 299 225 18

19 1126 0.0 1 773 244 109

20 1450 0.0 2 757 298 395

21 2132 0.0 2 796 878 458

22 3364 0.0 2 1550 893 921

23 4823 0.0 2 2797 1058 968

24 7616 0.0 2 4360 1631 1625

25 27578 0.0 2 15152 4206 8220

26 145155 0.0 2 46623 86510 12022

27 8329324 0.0 2 7989830 315267 24227

28 8490329 0.0 2 8140570 321411 28348

29 9180682 0.0 2 8796983 346527 37172

30 9293164 0.0 2 8799719 464686 28759

31 6221608 0.0 2 5958987 249989 12632

32 9144476 0.0 2 8898270 229597 16609

33 40450 0.0 2 11331 27591 1528

34 2074 0.0 2 813 288 973

35 923 0.0 2 575 253 95

36 446 0.0 2 331 77 38

37 453 0.0 2 291 102 60

38 268 0.0 2 215 23 30

39 216 0.0 2 170 29 17

40 240 0.0 2 134 28 78

41 251 0.0 2 102 45 104

42 151 0.0 2 122 12 17

43 107 0.0 2 80 17 10

44 113 0.0 2 94 9 10

45 98 0.0 2 64 15 19

46 84 0.0 2 61 18 5

47 98 0.0 2 85 8 5

48 84 0.0 2 67 9 8

49 80 0.0 2 69 8 3

50 74 0.0 2 62 8 4

51 70 0.0 2 56 8 6

52 88 0.0 2 68 15 5

53 64 0.0 2 53 7 4

54 52 0.0 2 41 8 3

55 61 0.0 2 47 8 6

56 46 0.0 2 33 10 3

57 57 0.0 2 44 8 5

58 31 0.0 2 26 4 1

59 37 0.0 2 28 7 2

60 32 0.0 2 27 4 1

61 24 0.0 2 16 5 3

62 35 0.0 2 23 4 8

63 23 0.0 2 15 4 4

64 25 0.0 2 18 3 4

65 24 0.0 2 19 3 2

66 36 0.0 2 9 24 3

67 18 0.0 2 16 1 1

68 9 0.0 2 7 2

69 11 0.0 2 8 0 3

70 6 0.0 2 3 3

71 27 0.0 2 8 18 1

72 13 0.0 2 8 2 3

73 8 0.0 2 8

74 17 0.0 2 7 7 3

75 7 0.0 2 5 1 1

76 6 0.0 2 2 2 2

77 10 0.0 2 4 1 5

78 13 0.0 2 4 6 3

79 5 0.0 2 3 2

80 5 0.0 2 1 2 2

81 9 0.0 2 5 2 2

82 3 0.0 2 2 1

83 4 0.0 2 1 3

84 3 0.0 2 1 2

87 1 0.0 2 0 1

88 1 0.0 2 0 0 1

89 2 0.0 2 0 0 2

90 1 0.0 2 0 1

91 1 0.0 2 0 1

92 2 0.0 2 0 1 1

93 1 0.0 2 0 0 1

94 1 0.0 2 0 0 1

97 2 0.0 2 0 0 2

99 1 0.0 2 0 1

101 3 0.0 2 0 2 1

102 2 0.0 2 0 0 2

103 4 0.0 2 0 0 4

104 1 0.0 2 0 0 1

106 2 0.0 2 0 0 2

107 2 0.0 2 0 0 2

108 8 0.0 2 0 5 3

109 1 0.0 2 0 0 1

110 2 0.0 2 0 2

111 3 0.0 2 0 2 1

112 3 0.0 2 0 0 3

113 14 0.0 2 0 4 10

114 8 0.0 2 0 2 6

115 28 0.0 2 0 20 8

116 14 0.0 2 0 9 5

117 7 0.0 2 0 5 2

118 6 0.0 2 0 4 2

119 4 0.0 2 0 3 1

120 9 0.0 2 0 6 3

121 4 0.0 2 0 2 2

122 8 0.0 2 0 0 8

125 1 0.0 2 0 0 1

127 2 0.0 2 0 0 2

128 1 0.0 2 0 0 1

129 1 0.0 2 0 0 1

139 1 0.0 2 0 0 1

140 1 0.0 2 0 0 1

143 1 0.0 2 0 1

145 1 0.0 2 1

146 1 0.0 2 0 1

151 1 0.0 2 0 0 1

152 2 0.0 2 0 1 1

153 1 0.0 2 0 0 1

160 1 0.0 2 0 1

162 1 0.0 2 0 0 1

165 1 0.0 2 0 0 1

172 1 0.0 2 0 1

175 1 0.0 2 0 0 1

189 1 0.0 2 0 0 1

207 1 0.0 2 0 1

209 1 0.0 2 0 0 1

226 1 0.0 2 0 0 1

228 1 0.0 2 0 0 1

230 1 0.0 2 0 0 1

231 1 0.0 2 0 0 1

250 7 0.0 2 0 0 7

270 1 0.0 2 0 1

271 2 0.0 2 2

272 2 0.0 2 1 1

273 3 0.0 2 3

274 10 0.0 2 9 1

275 11 0.0 2 10 1

276 6 0.0 2 6

277 2 0.0 2 2

278 37 0.0 2 0 9 28

279 1203 0.0 2 1 352 850

280 139 0.0 2 0 49 90

281 11 0.0 2 0 1 10

282 87 0.0 2 0 30 57

283 128 0.0 2 0 73 55

284 8 0.0 2 0 3 5

285 38 0.0 2 0 16 22

286 71 0.0 2 0 27 44

287 75 0.0 2 0 30 45

288 2 0.0 2 0 1 1

289 1 0.0 2 0 0 1

290 1 0.0 2 0 0 1

291 1 0.0 2 0 1

292 4 0.0 2 0 4

293 3 0.0 2 0 3

294 11 0.0 2 0 11

295 14 0.0 2 0 11 3

296 2 0.0 2 0 2

297 1 0.0 2 0 1

298 1 0.0 2 0 1

299 1 0.0 2 0 1

300 1 0.0 2 0 0 1

303 3 0.0 2 0 3

305 2 0.0 2 0 2

307 1 0.0 2 1

308 4 0.0 2 3 0 1

309 11 0.0 2 0 1 10

310 63 0.0 2 0 13 50

311 88 0.0 2 1 17 70

312 70 0.0 2 6 21 43

313 50 0.0 2 0 14 36

314 58 0.0 2 3 26 29

315 1 0.0 2 1

316 2 0.0 2 1 0 1

317 1 0.0 2 1

318 2 0.0 2 1 1

319 4 0.0 2 4

320 19 0.0 2 19

321 24 0.0 2 23 0 1

322 16 0.0 2 16

323 58 0.0 2 52 6

324 78 0.0 2 63 13 2

325 91 0.0 2 81 9 1

326 56 0.0 2 55 1

327 38 0.0 2 35 3

328 2 0.0 2 1 1

331 2 0.0 2 2

332 3 0.0 2 3

335 1 0.0 2 0 1

336 1 0.0 2 1

337 2 0.0 2 2

341 1 0.0 2 1

342 2 0.0 2 2

343 3 0.0 2 3

344 2 0.0 2 1 1

487 1 0.0 2 1

489 1 0.0 2 1

490 1 0.0 2 1

491 1 0.0 2 1

492 1 0.0 2 1

494 1 0.0 2 0 0 1

565 1 0.0 2 0 1

584 3 0.0 2 3

585 2 0.0 2 2

586 3 0.0 2 3

587 1 0.0 2 1

588 5 0.0 2 4 1

589 2 0.0 2 2

591 5 0.0 2 3 2

592 3 0.0 2 2 1

593 5 0.0 2 4 0 1

594 1 0.0 2 1

595 1 0.0 2 1

596 2 0.0 2 1 1

=== Adapter 2 ===

Sequence: ATTAGAGACCCCDGTAKTCCGT; Type: regular 3'; Length: 22; Trimmed: 30830 times.

No. of allowed errors:

0-9 bp: 0; 10-19 bp: 1; 20-22 bp: 2

Overview of removed sequences

length count expect max.err error counts

7 7 3112.6 0 7

9 3 194.5 0 2 1

10 1 48.6 1 0 1

11 1 12.2 1 0 1

22 2 0.0 2 0 1 1

24 1 0.0 2 0 0 1

25 130 0.0 2 67 28 35

26 1299 0.0 2 400 796 103

27 555 0.0 2 425 72 58

28 5789 0.0 2 5316 380 93

29 6445 0.0 2 6034 287 124

30 12462 0.0 2 11543 722 197

31 2257 0.0 2 1745 308 204

32 1836 0.0 2 1289 277 270

33 11 0.0 2 2 3 6

34 18 0.0 2 1 0 17

35 1 0.0 2 0 0 1

41 2 0.0 2 0 1 1

43 1 0.0 2 0 0 1

279 4 0.0 2 0 1 3

280 1 0.0 2 0 0 1

282 1 0.0 2 0 0 1

283 2 0.0 2 0 1 1

328 1 0.0 2 0 0 1

=== Adapter 3 ===

Sequence: GAGTGCCAGCMGCCGCGGTAA; Type: regular 5'; Length: 21; Trimmed: 49973622 times.

No. of allowed errors:

0-9 bp: 0; 10-19 bp: 1; 20-21 bp: 2

Overview of removed sequences

length count expect max.err error counts

3 1131 796825.2 0 1131

4 283 199206.3 0 283

5 60 49801.6 0 60

6 16 12450.4 0 16

7 10 3112.6 0 10

8 12 778.1 0 12

9 374 194.5 0 374

10 90 48.6 1 75 15

11 149 12.2 1 100 49

12 1255 3.0 1 1170 85

13 162 0.8 1 85 77

14 135 0.2 1 70 65

15 194 0.0 1 96 98

16 521 0.0 1 459 62

17 287 0.0 1 119 168

18 880 0.0 1 671 208 1

19 817 0.0 1 419 372 26

20 1719 0.0 2 1030 428 261

21 1849 0.0 2 788 557 504

22 2460 0.0 2 1227 656 577

23 3180 0.0 2 1810 686 684

24 4622 0.0 2 2769 956 897

25 5887 0.0 2 3628 1194 1065

26 10154 0.0 2 6811 1755 1588

27 23642 0.0 2 12194 3408 8040

28 148467 0.0 2 55498 82589 10380

29 7958205 0.0 2 7667378 278108 12719

30 8190967 0.0 2 7871397 301052 18518

31 8189288 0.0 2 7231714 283011 674563

32 7505287 0.0 2 7137235 337548 30504

33 11318675 0.0 2 10942279 353040 23356

34 6523272 0.0 2 6311260 191481 20531

35 56482 0.0 2 15202 16427 24853

36 15802 0.0 2 1252 173 14377

37 1004 0.0 2 800 90 114

38 680 0.0 2 579 43 58

39 617 0.0 2 491 38 88

40 386 0.0 2 342 23 21

41 281 0.0 2 240 20 21

42 224 0.0 2 197 19 8

43 171 0.0 2 138 23 10

44 133 0.0 2 107 14 12

45 136 0.0 2 108 14 14

46 109 0.0 2 92 9 8

47 122 0.0 2 111 6 5

48 152 0.0 2 80 8 64

49 116 0.0 2 85 4 27

50 103 0.0 2 78 7 18

51 108 0.0 2 91 2 15

52 104 0.0 2 67 3 34

53 78 0.0 2 62 3 13

54 86 0.0 2 81 2 3

55 65 0.0 2 64 1

56 73 0.0 2 68 3 2

57 303 0.0 2 53 5 245

58 82 0.0 2 37 2 43

59 47 0.0 2 41 1 5

60 51 0.0 2 44 1 6

61 59 0.0 2 48 3 8

62 42 0.0 2 35 6 1

63 38 0.0 2 34 1 3

64 38 0.0 2 33 4 1

65 24 0.0 2 22 0 2

66 31 0.0 2 27 0 4

67 32 0.0 2 28 2 2

68 20 0.0 2 16 0 4

69 14 0.0 2 11 1 2

70 28 0.0 2 22 3 3

71 13 0.0 2 10 0 3

72 16 0.0 2 13 0 3

73 7 0.0 2 5 0 2

74 13 0.0 2 11 1 1

75 18 0.0 2 13 1 4

76 13 0.0 2 9 0 4

77 8 0.0 2 5 0 3

78 10 0.0 2 7 0 3

79 11 0.0 2 11

80 13 0.0 2 10 0 3

81 8 0.0 2 6 0 2

82 5 0.0 2 3 1 1

83 10 0.0 2 6 1 3

84 4 0.0 2 3 0 1

85 11 0.0 2 9 0 2

86 5 0.0 2 1 0 4

87 2 0.0 2 2

88 7 0.0 2 2 0 5

89 3 0.0 2 0 0 3

90 1 0.0 2 1

91 2 0.0 2 0 0 2

92 1 0.0 2 0 0 1

93 2 0.0 2 2

94 4 0.0 2 1 0 3

95 4 0.0 2 0 0 4

97 1 0.0 2 0 0 1

100 1 0.0 2 0 0 1

101 1 0.0 2 0 0 1

106 2 0.0 2 0 0 2

110 2 0.0 2 0 0 2

111 1 0.0 2 0 0 1

112 2 0.0 2 0 0 2

113 3 0.0 2 0 0 3

114 4 0.0 2 0 0 4

116 1 0.0 2 0 0 1

117 1 0.0 2 0 0 1

125 3 0.0 2 0 0 3

126 2 0.0 2 0 0 2

127 2 0.0 2 0 0 2

128 1 0.0 2 0 0 1

129 3 0.0 2 0 0 3

130 5 0.0 2 0 0 5

131 13 0.0 2 0 0 13

132 10 0.0 2 0 0 10

133 17 0.0 2 0 0 17

134 6 0.0 2 0 0 6

135 14 0.0 2 0 0 14

136 10 0.0 2 0 0 10

137 3 0.0 2 0 0 3

138 4 0.0 2 0 0 4

140 1 0.0 2 0 0 1

141 1 0.0 2 0 0 1

142 2 0.0 2 0 0 2

144 1 0.0 2 0 0 1

145 1 0.0 2 0 0 1

146 1 0.0 2 0 0 1

147 2 0.0 2 0 0 2

148 2 0.0 2 0 0 2

149 1 0.0 2 0 0 1

150 1 0.0 2 0 0 1

151 2 0.0 2 0 0 2

152 3 0.0 2 0 0 3

153 7 0.0 2 0 0 7

154 6 0.0 2 0 0 6

155 7 0.0 2 0 0 7

156 16 0.0 2 0 0 16

157 8 0.0 2 0 1 7

158 18 0.0 2 0 2 16

159 9 0.0 2 0 0 9

160 10 0.0 2 0 0 10

161 3 0.0 2 0 0 3

162 1 0.0 2 0 0 1

163 1 0.0 2 0 0 1

164 1 0.0 2 0 0 1

171 1 0.0 2 0 0 1

172 1 0.0 2 0 0 1

173 5 0.0 2 0 0 5

174 1 0.0 2 0 0 1

175 1 0.0 2 0 0 1

176 1 0.0 2 0 0 1

177 1 0.0 2 0 0 1

179 1 0.0 2 0 0 1

180 10 0.0 2 0 0 10

183 1 0.0 2 0 0 1

191 1 0.0 2 0 0 1

194 1 0.0 2 0 0 1

195 3 0.0 2 0 0 3

196 2 0.0 2 0 0 2

197 2 0.0 2 0 0 2

198 1 0.0 2 0 0 1

199 1 0.0 2 0 0 1

200 1 0.0 2 0 0 1

201 1 0.0 2 0 0 1

203 1 0.0 2 0 0 1

241 4 0.0 2 2 2

242 12 0.0 2 0 11 1

243 20 0.0 2 3 16 1

244 46 0.0 2 5 38 3

245 62 0.0 2 9 44 9

259 2 0.0 2 0 2

271 1 0.0 2 0 1

287 1 0.0 2 0 0 1

288 1 0.0 2 0 1

290 3 0.0 2 0 3

291 19 0.0 2 5 6 8

292 64 0.0 2 4 50 10

293 135 0.0 2 23 90 22

294 333 0.0 2 45 231 57

295 469 0.0 2 60 339 70

296 3 0.0 2 0 0 3

297 1 0.0 2 0 0 1

323 1 0.0 2 0 0 1

326 1 0.0 2 0 1

327 1 0.0 2 0 1

329 1 0.0 2 1

394 2 0.0 2 1 0 1

431 1 0.0 2 0 0 1

449 1 0.0 2 1

450 2 0.0 2 1 1

451 1 0.0 2 0 0 1

452 2 0.0 2 1 1

463 1 0.0 2 0 0 1

546 4 0.0 2 0 3 1

547 4 0.0 2 0 3 1

548 6 0.0 2 0 6

549 4 0.0 2 1 3

550 5 0.0 2 1 3 1

551 12 0.0 2 1 8 3

552 5 0.0 2 1 4

553 3 0.0 2 1 2

554 6 0.0 2 3 3

555 4 0.0 2 1 3

556 1 0.0 2 0 0 1

557 1 0.0 2 1

560 1 0.0 2 0 1

561 5 0.0 2 3 2

562 1 0.0 2 1

563 9 0.0 2 5 4

564 9 0.0 2 4 5

565 3 0.0 2 1 2

566 6 0.0 2 0 4 2

567 2 0.0 2 0 2

580 1 0.0 2 1

581 2 0.0 2 0 1 1

595 1 0.0 2 1

596 1 0.0 2 1

=== Adapter 4 ===

Sequence: GAGTGCCAGCMGCWGCGGTAA; Type: regular 5'; Length: 21; Trimmed: 39998 times.

No. of allowed errors:

0-9 bp: 0; 10-19 bp: 1; 20-21 bp: 2

Overview of removed sequences

length count expect max.err error counts

8 3 778.1 0 3

9 7 194.5 0 4 3

10 6 48.6 1 3 3

11 3 12.2 1 0 3

12 1 3.0 1 1

13 1 0.8 1 1

16 1 0.0 1 1

18 1 0.0 1 1

21 1 0.0 2 1

22 4 0.0 2 3 1

23 1 0.0 2 1

25 5 0.0 2 4 1

26 10 0.0 2 4 5 1

27 12 0.0 2 7 1 4

28 172 0.0 2 37 113 22

29 11789 0.0 2 11242 463 84

30 5650 0.0 2 5343 256 51

31 5765 0.0 2 4691 228 846

32 4971 0.0 2 4225 675 71

33 7070 0.0 2 6732 273 65

34 4465 0.0 2 4196 213 56

35 34 0.0 2 5 10 19

36 13 0.0 2 0 0 13

37 2 0.0 2 1 0 1

38 2 0.0 2 2

40 1 0.0 2 0 0 1

42 1 0.0 2 0 1

64 1 0.0 2 1

100 1 0.0 2 0 0 1

293 1 0.0 2 1

294 1 0.0 2 0 1

295 2 0.0 2 0 1 1

592 1 0.0 2 0 0 1

=== Adapter 5 ===

Sequence: GAGTGCCAGCMGCCGCGGTCA; Type: regular 5'; Length: 21; Trimmed: 22971 times.

No. of allowed errors:

0-9 bp: 0; 10-19 bp: 1; 20-21 bp: 2

Overview of removed sequences

length count expect max.err error counts

3 814 796825.2 0 814

4 226 199206.3 0 226

5 61 49801.6 0 61

6 1 12450.4 0 1

9 1 194.5 0 1

11 2 12.2 1 1 1

12 5 3.0 1 4 1

13 1 0.8 1 1

14 2 0.2 1 2

15 1 0.0 1 0 1

16 10 0.0 1 10

17 4 0.0 1 2 2

18 14 0.0 1 12 2

19 21 0.0 1 15 6

20 10 0.0 2 0 5 5

21 29 0.0 2 0 14 15

22 23 0.0 2 0 12 11

23 49 0.0 2 0 23 26

24 55 0.0 2 0 23 32

25 104 0.0 2 0 57 47

26 92 0.0 2 0 51 41

27 478 0.0 2 0 170 308

28 4658 0.0 2 6 4532 120

29 13010 0.0 2 1007 11761 242

30 1608 0.0 2 12 1500 96

31 596 0.0 2 7 280 309

32 397 0.0 2 13 304 80

33 412 0.0 2 16 361 35

34 236 0.0 2 10 203 23

35 13 0.0 2 0 4 9

36 6 0.0 2 0 1 5

37 1 0.0 2 0 0 1

38 2 0.0 2 0 2

133 1 0.0 2 0 0 1

176 1 0.0 2 0 0 1

243 1 0.0 2 1

294 6 0.0 2 4 0 2

295 1 0.0 2 1

560 1 0.0 2 1

561 4 0.0 2 4

562 2 0.0 2 1 0 1

563 5 0.0 2 4 1

564 3 0.0 2 1 2

565 1 0.0 2 1

566 2 0.0 2 2

591 1 0.0 2 0 0 1

=== Adapter 6 ===

Sequence: GAGTGKCAGCMGCCGCGGTAA; Type: regular 5'; Length: 21; Trimmed: 900555 times.

No. of allowed errors:

0-9 bp: 0; 10-19 bp: 1; 20-21 bp: 2

Overview of removed sequences

length count expect max.err error counts

15 4 0.0 1 0 4

16 97 0.0 1 42 55

17 87 0.0 1 26 61

18 72 0.0 1 17 55

19 72 0.0 1 7 46 19

20 77 0.0 2 19 24 34

21 77 0.0 2 11 18 48

22 88 0.0 2 22 16 50

23 125 0.0 2 30 19 76

24 140 0.0 2 42 21 77

25 186 0.0 2 53 29 104

26 244 0.0 2 91 34 119

27 292 0.0 2 124 58 110

28 785 0.0 2 394 274 117

29 27798 0.0 2 26700 908 190

30 29673 0.0 2 27897 1139 637

31 39580 0.0 2 29145 8707 1728

32 735381 0.0 2 713809 21093 479

33 42004 0.0 2 38152 3669 183

34 23321 0.0 2 21809 576 936

35 213 0.0 2 81 46 86

36 81 0.0 2 25 2 54

37 10 0.0 2 8 1 1

38 17 0.0 2 14 3

39 17 0.0 2 12 5

40 3 0.0 2 3

41 7 0.0 2 6 1

42 9 0.0 2 8 0 1

43 9 0.0 2 3 5 1

44 1 0.0 2 0 1

45 2 0.0 2 2

46 3 0.0 2 3

47 1 0.0 2 1

48 2 0.0 2 2

49 1 0.0 2 1

50 8 0.0 2 7 1

51 9 0.0 2 9

52 5 0.0 2 5

53 2 0.0 2 2

55 3 0.0 2 3

56 3 0.0 2 2 1

58 1 0.0 2 1

64 1 0.0 2 1

66 1 0.0 2 1

71 2 0.0 2 2

89 3 0.0 2 3

97 1 0.0 2 0 1

98 4 0.0 2 0 4

99 1 0.0 2 0 1

100 3 0.0 2 0 3

101 2 0.0 2 0 2

153 2 0.0 2 0 2

245 1 0.0 2 0 1

292 1 0.0 2 0 1

293 4 0.0 2 0 2 2

294 8 0.0 2 1 5 2

295 9 0.0 2 2 5 2

447 1 0.0 2 0 1

561 1 0.0 2 0 0 1

=== Adapter 7 ===

Sequence: GAGTGGCAGYCGCCRCGGKAA; Type: regular 5'; Length: 21; Trimmed: 2162 times.

No. of allowed errors:

0-9 bp: 0; 10-19 bp: 1; 20-21 bp: 2

Overview of removed sequences

length count expect max.err error counts

3 969 796825.2 0 969

4 228 199206.3 0 228

5 64 49801.6 0 64

6 5 12450.4 0 5

7 9 3112.6 0 9

8 1 778.1 0 1

10 1 48.6 1 0 1

11 2 12.2 1 0 2

12 38 3.0 1 28 10

13 13 0.8 1 1 12

14 2 0.2 1 0 2

15 5 0.0 1 2 3

23 1 0.0 2 0 0 1

24 1 0.0 2 0 0 1

28 5 0.0 2 0 1 4

29 22 0.0 2 0 15 7

30 39 0.0 2 1 29 9

31 26 0.0 2 3 19 4

32 295 0.0 2 253 32 10

33 419 0.0 2 382 31 6

34 15 0.0 2 0 12 3

35 1 0.0 2 0 1

595 1 0.0 2 0 1

WARNING:

    One or more of your adapter sequences may be incomplete.

    Please see the detailed output above.

##Summarize

usearch7 -fastq_stats Trimmed_Rev.fastq -log stats.log

21:43  38Mb  100.0% Reading Trimmed_Rev.fastq

  50983994  Recs (51.0M), 0 too long

     252.9  Avg length

     12.9G  Bases

##Filter reads

usearch7 -fastq_filter Trimmed_Rev.fastq -fastaout seqs.filtered.fasta -fastq_maxee 0.5 -threads 24

15:20 2.4Mb  100.0% Converting, 51.0M recs, 48390704 converted (94.9%)

  50983994  FASTQ recs (51.0M)

   2593280  Low qual recs discarded (expected errs > 0.50)

  48390704  Converted (48.4M, 94.9%)

##Dereplicate sequences

usearch7 -derep_fulllength seqs.filtered.fasta -output seqs.filtered.derep.fasta -sizeout -threads 24

04:34 15.7Gb  100.0% Reading seqs.filtered.fasta

850 short sequences length < 32 discarded

06:50 18.0Gb 3593159 (3.6M) uniques, avg cluster 13.5, median 1, max 16739782

07:37 18.0Gb  100.0% Writing seqs.filtered.derep.fasta

##Remove singletons

usearch7 -sortbysize seqs.filtered.derep.fasta -minsize 2 -output seqs.filtered.derep.mc2.fasta

00:00 2.4Mb Reading seqs.filtered.derep.fasta, 1.0Gb

00:03 1.0Gb 3593159 (3.6M) seqs, min 33, avg 254, max 489nt

00:07 1.1Gb Getting sizes

00:14 1.1Gb Sorting 871827 sequences

00:18 1.1Gb  100.0% Writing seqs.filtered.derep.mc2.fasta

##Cluster OTUs

usearch7 -cluster_otus seqs.filtered.derep.mc2.fasta -otus seqs.filtered.derep.mc2.repset.fasta

59:02 1.4Gb  100.0% 36766 OTUs

Input seqs  871827 (871.8k)

      OTUs  36766 (36.8k)

   Members  794573 (794.6k)

  Chimeras  40488 (40.5k)

   Max mem  1.4Gb

      Time  59:02

Throughput  246.1 seqs/sec.

usearch7 -uchime_ref seqs.filtered.derep.mc2.repset.fasta -db /data1/qiime/qiime_1.9_base/lib/python2.7/site-packages/qiime_default_reference/gg_13_8_otus/rep_set/97_otus.fasta -minh 1.0 -strand plus -nonchimeras seqs.filtered.derep.mc2.repset.nochimeras.fasta -threads 24

Licensed to: robesonms@ornl.gov

00:00 2.4Mb Reading seqs.filtered.derep.mc2.repset.fasta, 11Mb

00:00  13Mb 36766 (36.8k) seqs, min 158, avg 255, max 485nt

00:00  14Mb Reading /data1/qiime/qiime_1.9_base/lib/python2.7/site-packages/qiime_default_reference/gg_13_8_otus/rep_set/97_otus.fasta, 143Mb

00:00 157Mb 99322 (99.3k) seqs, min 1254, avg 1433, max 2353nt

00:06 159Mb  100.0% Masking

00:16 159Mb  100.0% Word stats

00:16 697Mb  100.0% Building slots

00:37 697Mb  100.0% Build index

01:18 1.8Gb  100.0% Search 20/36766 chimeras found (0.1%)

01:18 1.8Gb  100.0% Writing 36746 non-chimeras

python /data2/m3q/Python_scripts/fasta_number.py seqs.filtered.derep.mc2.repset.nochimeras.fasta OTU_ > seqs.filtered.derep.mc2.repset.nochimeras.OTUs.fasta

usearch7 -usearch_global seqs.filtered.fasta -db seqs.filtered.derep.mc2.repset.nochimeras.OTUs.fasta -strand plus -id 0.97 -uc otu.map.uc -threads 24

00:00  19Mb Reading seqs.filtered.derep.mc2.repset.nochimeras.OTUs.fasta, 9.9Mb

00:00  29Mb 36743 (36.7k) seqs, min 158, avg 255, max 485nt

00:00  30Mb  100.0% Masking

00:01  30Mb  100.0% Word stats

00:01  67Mb  100.0% Building slots

00:02  67Mb  100.0% Build index

00:38 1.0Gb    3.9% Searching, 97.9% matched

fastaseqsource.cpp(207):

WARNING: Short sequence(s) discarded, min seq length is 32

16:22 1.0Gb  100.0% Searching, 98.6% matched

python /data2/m3q/Python_scripts/uc2otutab_mod.py otu.map.uc > seqs.filtered.derep.mc2.repset.nochimeras.OTU-table.txt

a

##copy to local machine for taxonomy assignment via blast

parallel_assign_taxonomy_blast.py -i Desktop/PMIFinalAnalysisAug2016/seqs.filtered.derep.mc2.repset.nochimeras.OTUs.fasta -o Desktop/PMIFinalAnalysisAug2016/Blast_taxonomy/ -O 10

biom convert -i seqs.filtered.derep.mc2.repset.nochimeras.OTU-table.txt --table-type="OTU table" -o AtlasBacterialRawOTUTable.biom --to-hdf5

biom add-metadata --sc-separated taxonomy --observation-header OTUID,taxonomy --observation-metadata-fp Blast_taxonomy/seqs.filtered.derep.mc2.repset.nochimeras.OTUs_tax_assignments.txt -i AtlasBacterialRawOTUTable.biom -o AtlasBacterialRawOTUTablewTax.biom

biom convert -i AtlasBacterialRawOTUTablewTax.biom -o AtlasBacterialRawOTUTablewtax.txt --to-tsv --header-key taxonomy

Fungal Data Processing:

*###ITS2 analysis for PMI-ATLAS dataset, 26July2016###

###each run had R1, R2 runs joined individually. This uses the default of fastq-join, example below, done for each of 6 runs, but had to redo for first run as directory was deleted###

ava@orion:/data2/ava/atlas_fungal_run1$ join_paired_ends.py -f Undetermined_S0_L001_R1_001.fastq -r Undetermined_S0_L001_R2_001.fastq -b Undetermined_S0_L001_I1_001.fastq -o joined_ends

###demultiplex using a stringent sequence quality threshold, only 1 run is given as an example###

localadmin:~ localadmin$ split_libraries_fastq.py -i /Volumes/Veeach/All_Sequences/ORNL/ATLAS/Demultiplexed_Run3/Joined_Ends/fastqjoin.join.fastq -b /Volumes/Veeach/All_Sequences/ORNL/ATLAS/Demultiplexed_Run3/Joined_Ends/fastqjoin.join_barcodes.fastq -m /Volumes/Veeach/All_Sequences/ORNL/ATLAS/Run3/ATLAS_Fungi_Run3_PMIonly_MAP.txt --store_demultiplexed_fastq --rev_comp_mapping_barcodes -q 19 -o /Volumes/Veeach/All_Sequences/ORNL/ATLAS/Demultiplexed_Run3/Split_libraries

###concatenate all 6 fasta files###

MacQIIME localadmin:~ $ cat /Volumes/Veeach/All_Sequences/ORNL/ATLAS/Demultiplexed_PNAS/Split_libraries/seqs.fastq /Volumes/Veeach/All_Sequences/ORNL/ATLAS/Demultiplexed_Run1/split_libraries/seqs.fastq /Volumes/Veeach/All_Sequences/ORNL/ATLAS/Demultiplexed_Run2/Split_libraries/seqs.fastq /Volumes/Veeach/All_Sequences/ORNL/ATLAS/Demultiplexed_Run3/Split_libraries/seqs.fastq /Volumes/Veeach/All_Sequences/ORNL/ATLAS/Demultiplexed_Run4/Split_libraries/seqs.fastq /Volumes/Veeach/All_Sequences/ORNL/ATLAS/Demultiplexed_Run5/Split_Libraries/seqs.fastq > /Volumes/Veeach/All_Sequences/ORNL/ATLAS/all_ATLAS_ITS2_seqs.fastq

###cut off primers and adapters###

Trimming 9 adapter(s) with at most 10.0% errors in single-end mode ...

Finished in 4804.02 s (264 us/read; 0.23 M reads/minute).

=== Summary ===

Total reads processed:              18,225,926

Reads with adapters:                17,688,604 (97.1%)

Reads written (passing filters):    17,688,604 (97.1%)

Total basepairs processed: 6,927,397,110 bp

Total written (filtered):  6,210,262,674 bp (89.6%)

=== Adapter 1 ===

Sequence: TCCTSCGCTTATTGATATGC; Type: regular 3'; Length: 20; Trimmed: 12932 times.

No. of allowed errors:

0-9 bp: 0; 10-19 bp: 1; 20 bp: 2

Bases preceding removed adapters:

  A: 14.3%

  C: 12.7%

  G: 55.1%

  T: 17.9%

  none/other: 0.0%

Overview of removed sequences

length count expect max.err error counts

3 8858 284780.1 0 8858

4 2917 71195.0 0 2917

5 657 17798.8 0 657

6 205 4449.7 0 205

7 71 1112.4 0 71

8 33 278.1 0 33

10 140 17.4 1 0 140

11 47 4.3 1 0 47

12 4 1.1 1 0 4

=== Adapter 2 ===

Sequence: TCCTCGCCTTATTGATATGC; Type: regular 3'; Length: 20; Trimmed: 32 times.

No. of allowed errors:

0-9 bp: 0; 10-19 bp: 1; 20 bp: 2

Bases preceding removed adapters:

  A: 31.2%

  C: 18.8%

  G: 46.9%

  T: 3.1%

  none/other: 0.0%

Overview of removed sequences

length count expect max.err error counts

6 23 4449.7 0 23

7 7 1112.4 0 7

11 1 4.3 1 0 1

12 1 1.1 1 0 1

=== Adapter 3 ===

Sequence: CATCGATGAAGAACGCAG; Type: regular 5'; Length: 18; Trimmed: 13408039 times.

No. of allowed errors:

0-9 bp: 0; 10-18 bp: 1

Overview of removed sequences

length count expect max.err error counts

3 9727 284780.1 0 9727

4 2198 71195.0 0 2198

5 507 17798.8 0 507

6 149 4449.7 0 149

7 174 1112.4 0 174

8 34 278.1 0 34

9 78 69.5 0 38 40

10 156 17.4 1 132 24

11 177 4.3 1 115 62

12 207 1.1 1 144 63

13 557 0.3 1 460 97

14 491 0.1 1 285 206

15 902 0.0 1 641 261

16 665 0.0 1 453 212

17 834 0.0 1 383 451

18 1216 0.0 1 688 528

19 1526 0.0 1 945 581

20 2619 0.0 1 1948 671

21 3853 0.0 1 2961 892

22 5350 0.0 1 4249 1101

23 7682 0.0 1 6283 1399

24 10067 0.0 1 8190 1877

25 15097 0.0 1 12675 2422

26 21156 0.0 1 18454 2702

27 57521 0.0 1 32660 24861

28 1941755 0.0 1 1890393 51362

29 2350570 0.0 1 2283010 67560

30 3675070 0.0 1 3605355 69715

31 2168194 0.0 1 2117819 50375

32 1976692 0.0 1 1935097 41595

33 1150398 0.0 1 1132122 18276

34 1843 0.0 1 1548 295

35 262 0.0 1 252 10

36 111 0.0 1 107 4

37 75 0.0 1 75

38 36 0.0 1 35 1

39 25 0.0 1 24 1

40 16 0.0 1 16

41 8 0.0 1 7 1

42 7 0.0 1 5 2

43 4 0.0 1 4

44 1 0.0 1 1

45 1 0.0 1 1

50 2 0.0 1 1 1

51 1 0.0 1 1

52 2 0.0 1 2

53 1 0.0 1 1

54 1 0.0 1 1

55 2 0.0 1 2

57 1 0.0 1 1

63 1 0.0 1 1

66 3 0.0 1 3

67 1 0.0 1 1

68 1 0.0 1 1

69 1 0.0 1 1

71 1 0.0 1 1

74 1 0.0 1 1

78 2 0.0 1 2

79 1 0.0 1 1

88 1 0.0 1 1

117 1 0.0 1 1

127 1 0.0 1 1

269 1 0.0 1 1

288 1 0.0 1 1

347 1 0.0 1 1

=== Adapter 4 ===

Sequence: CAACGATGAAGAACGCAG; Type: regular 5'; Length: 18; Trimmed: 1676697 times.

No. of allowed errors:

0-9 bp: 0; 10-18 bp: 1

Overview of removed sequences

length count expect max.err error counts

15 9 0.0 1 0 9

16 137 0.0 1 96 41

17 122 0.0 1 67 55

18 187 0.0 1 76 111

19 249 0.0 1 142 107

20 360 0.0 1 228 132

21 611 0.0 1 459 152

22 856 0.0 1 643 213

23 1198 0.0 1 978 220

24 1807 0.0 1 1510 297

25 2525 0.0 1 2176 349

26 3717 0.0 1 3320 397

27 17690 0.0 1 9069 8621

28 1621667 0.0 1 1599810 21857

29 8349 0.0 1 7689 660

30 6778 0.0 1 6509 269

31 4135 0.0 1 3921 214

32 4085 0.0 1 3907 178

33 2041 0.0 1 1939 102

34 116 0.0 1 73 43

35 17 0.0 1 12 5

36 4 0.0 1 4

37 26 0.0 1 26

38 3 0.0 1 3

39 1 0.0 1 1

40 1 0.0 1 1

43 1 0.0 1 1

47 1 0.0 1 1

49 1 0.0 1 0 1

55 2 0.0 1 2

134 1 0.0 1 0 1

=== Adapter 5 ===

Sequence: CACCGATGAAGAACGCAG; Type: regular 5'; Length: 18; Trimmed: 1247113 times.

No. of allowed errors:

0-9 bp: 0; 10-18 bp: 1

Overview of removed sequences

length count expect max.err error counts

16 100 0.0 1 88 12

17 160 0.0 1 88 72

18 159 0.0 1 78 81

19 252 0.0 1 159 93

20 397 0.0 1 266 131

21 559 0.0 1 397 162

22 765 0.0 1 467 298

23 924 0.0 1 665 259

24 1181 0.0 1 927 254

25 1710 0.0 1 1432 278

26 2245 0.0 1 2012 233

27 3115 0.0 1 2769 346

28 21373 0.0 1 14710 6663

29 1185540 0.0 1 1167806 17734

30 12254 0.0 1 11582 672

31 6517 0.0 1 6262 255

32 6493 0.0 1 6218 275

33 3267 0.0 1 3204 63

34 47 0.0 1 44 3

35 18 0.0 1 17 1

36 8 0.0 1 8

37 7 0.0 1 7

38 8 0.0 1 8

39 7 0.0 1 7

46 1 0.0 1 1

49 1 0.0 1 1

53 1 0.0 1 1

62 1 0.0 1 1

81 3 0.0 1 3

=== Adapter 6 ===

Sequence: CACCGATGAAGAACGCAG; Type: regular 5'; Length: 18; Trimmed: 0 times.

=== Adapter 7 ===

Sequence: CATCGATGAAGAACGTAG; Type: regular 5'; Length: 18; Trimmed: 564251 times.

No. of allowed errors:

0-9 bp: 0; 10-18 bp: 1

Overview of removed sequences

length count expect max.err error counts

3 7477 284780.1 0 7477

4 1890 71195.0 0 1890

5 244 17798.8 0 244

6 18 4449.7 0 18

7 5 1112.4 0 5

9 42 69.5 0 0 42

10 1 17.4 1 0 1

11 3 4.3 1 3

13 10 0.3 1 10

14 6 0.1 1 5 1

15 24 0.0 1 22 2

16 12 0.0 1 10 2

17 19 0.0 1 15 4

18 50 0.0 1 32 18

19 59 0.0 1 42 17

20 98 0.0 1 79 19

21 179 0.0 1 132 47

22 187 0.0 1 169 18

23 260 0.0 1 222 38

24 407 0.0 1 353 54

25 580 0.0 1 518 62

26 881 0.0 1 779 102

27 1298 0.0 1 1160 138

28 7971 0.0 1 7576 395

29 12926 0.0 1 10684 2242

30 499813 0.0 1 495067 4746

31 17156 0.0 1 16253 903

32 8379 0.0 1 7910 469

33 4125 0.0 1 3959 166

34 52 0.0 1 52

35 38 0.0 1 38

36 12 0.0 1 12

38 3 0.0 1 3

39 6 0.0 1 6

40 1 0.0 1 1

49 1 0.0 1 1

51 3 0.0 1 3

52 3 0.0 1 3

53 5 0.0 1 5

58 4 0.0 1 4

76 1 0.0 1 1

77 2 0.0 1 2

=== Adapter 8 ===

Sequence: CATCGATGAAGAACGTGG; Type: regular 5'; Length: 18; Trimmed: 248433 times.

No. of allowed errors:

0-9 bp: 0; 10-18 bp: 1

Overview of removed sequences

length count expect max.err error counts

3 10675 284780.1 0 10675

4 2248 71195.0 0 2248

5 423 17798.8 0 423

6 71 4449.7 0 71

7 17 1112.4 0 17

8 3 278.1 0 3

9 2 69.5 0 0 2

10 32 17.4 1 0 32

12 2 1.1 1 2

13 1 0.3 1 1

14 3 0.1 1 3

15 12 0.0 1 10 2

16 6 0.0 1 5 1

17 12 0.0 1 10 2

18 20 0.0 1 11 9

19 23 0.0 1 19 4

20 36 0.0 1 30 6

21 76 0.0 1 56 20

22 114 0.0 1 91 23

23 147 0.0 1 128 19

24 167 0.0 1 143 24

25 260 0.0 1 238 22

26 354 0.0 1 325 29

27 546 0.0 1 482 64

28 1736 0.0 1 1439 297

29 1984 0.0 1 1745 239

30 5235 0.0 1 4184 1051

31 221474 0.0 1 219485 1989

32 1748 0.0 1 1677 71

33 981 0.0 1 567 414

34 18 0.0 1 15 3

35 5 0.0 1 5

36 1 0.0 1 1

38 1 0.0 1 1

=== Adapter 9 ===

Sequence: CATCGATGAAGAACGCTG; Type: regular 5'; Length: 18; Trimmed: 531107 times.

No. of allowed errors:

0-9 bp: 0; 10-18 bp: 1

Overview of removed sequences

length count expect max.err error counts

3 9236 284780.1 0 9236

4 2867 71195.0 0 2867

5 595 17798.8 0 595

6 216 4449.7 0 216

7 107 1112.4 0 107

9 78 69.5 0 0 78

10 52 17.4 1 1 51

11 1 4.3 1 1

12 5 1.1 1 5

13 4 0.3 1 4

14 5 0.1 1 2 3

15 8 0.0 1 7 1

16 6 0.0 1 5 1

17 7 0.0 1 6 1

18 21 0.0 1 12 9

19 32 0.0 1 29 3

20 86 0.0 1 69 17

21 97 0.0 1 88 9

22 101 0.0 1 76 25

23 251 0.0 1 204 47

24 295 0.0 1 260 35

25 374 0.0 1 347 27

26 587 0.0 1 513 74

27 1077 0.0 1 997 80

28 4441 0.0 1 4190 251

29 5323 0.0 1 5068 255

30 7254 0.0 1 6964 290

31 10825 0.0 1 8495 2330

32 484601 0.0 1 480529 4072

33 2492 0.0 1 2343 149

34 39 0.0 1 39

35 10 0.0 1 10

36 8 0.0 1 8

37 3 0.0 1 3

38 1 0.0 1 1

41 2 0.0 1 2

usearch v7.0.1001_i86linux64, 264Gb RAM, 48 cores

(C) Copyright 2013 Robert C. Edgar, all rights reserved.

http://drive5.com

Licensed to: robesonms@ornl.gov

09:27  38Mb  100.0% Reading ITS2_trimmed.fastq

  17688604  Recs (17.7M), 0 too long

     351.1  Avg length

      6.2G  Bases

###summarize###

ava@orion:/data2/ava/fungal_analysis_ATLAS$ usearch7 -fastq_stats trimmed.fastq -log stats.log

usearch v7.0.1001_i86linux64, 264Gb RAM, 48 cores

(C) Copyright 2013 Robert C. Edgar, all rights reserved.

http://drive5.com

Licensed to: robesonms@ornl.gov

09:36  38Mb  100.0% Reading trimmed.fastq

  17688604  Recs (17.7M), 0 too long

     351.1  Avg length

      6.2G  Bases

ava@orion:/data2/ava/fungal_analysis_ATLAS$

###Filter reads - truncate to 200 bp, and remove reads with > 0 expected error probability###

ava@orion:/data2/ava/fungal_analysis_ATLAS$ usearch7 -fastq_filter trimmed.fastq -fastaout trimmed_filtered.fasta -fastqout trimmed_filtered.fastq -fastq_trunclen 200 -fastq_minlen 200 -fastq_maxee 0.5

usearch v7.0.1001_i86linux64, 264Gb RAM, 48 cores

(C) Copyright 2013 Robert C. Edgar, all rights reserved.

http://drive5.com

Licensed to: robesonms@ornl.gov

06:50 2.4Mb  100.0% Converting, 17.7M recs, 17413260 converted (98.4%)

  17688604  FASTQ recs (17.7M)

         3  Short recs discarded (<200)

    275341  Low qual recs discarded (expected errs > 0.50)

  17413260  Converted (17.4M, 98.4%)

###Dereplicate###

ava@orion:/data2/ava/fungal_analysis_ATLAS$ usearch7 -derep_fulllength trimmed_filtered.fasta -output derep_ITS2.fasta -sizeout -threads 24

usearch v7.0.1001_i86linux64, 264Gb RAM, 48 cores

(C) Copyright 2013 Robert C. Edgar, all rights reserved.

http://drive5.com

Licensed to: robesonms@ornl.gov

01:20 4.5Gb  100.0% Reading trimmed_filtered.fasta

01:55 5.7Gb 633239 (633.2k) uniques, avg cluster 27.5, median 1, max 1899333

02:02 5.7Gb  100.0% Writing derep_ITS2.fasta

###sort by sequence abundance###

ava@orion:/data2/ava/fungal_analysis_ATLAS$ usearch7 -sortbysize derep_ITS2.fasta -minsize 2 -output derep_nosingle_ITS2.fasta

usearch v7.0.1001_i86linux64, 264Gb RAM, 48 cores

(C) Copyright 2013 Robert C. Edgar, all rights reserved.

http://drive5.com

Licensed to: robesonms@ornl.gov

00:00 2.4Mb Reading derep_ITS2.fasta, 144Mb

00:01 146Mb 633239 (633.2k) seqs, min 200, avg 200, max 200nt

00:01 159Mb Getting sizes

00:02 164Mb Sorting 197780 sequences

00:03 165Mb  100.0% Writing derep_nosingle_ITS2.fasta

###cluster using usearch###

ava@orion:/data2/ava/fungal_analysis_ATLAS$ usearch7 -cluster_otus derep_nosingle_nochimeras_ITS2.fasta -otus atlas_repset.fasta

usearch v7.0.1001_i86linux64, 264Gb RAM, 48 cores

(C) Copyright 2013 Robert C. Edgar, all rights reserved.

http://drive5.com

Licensed to: robesonms@ornl.gov

26:16 436Mb  100.0% 10301 OTUs

Input seqs  197756 (197.8k)

      OTUs  10301 (10.3k)

   Members  182157 (182.2k)

  Chimeras  5298

   Max mem  436Mb

      Time  26:16

Throughput  125.5 seqs/sec.

###copy over reference database UNITE into current directory###

ava@orion:/data2/ava$ scp /data2/ava/atlas_fungal_run2/sh_refs_qiime_ver7_97_31.01.2016.fasta /data2/ava/fungal_analysis_ATLAS

ava@orion:/data2/ava$ scp /data2/ava/atlas_fungal_run2/sh_taxonomy_qiime_ver7_97_31.01.2016.txt /data2/ava/fungal_analysis_ATLAS

###uchime chimera detection and separation###

ava@orion:/data2/ava/fungal_analysis_ATLAS$ usearch7 -uchime_ref atlas_repset.fasta -minh 1 -db sh_refs_qiime_ver7_97_31.01.2016.fasta -strand plus -nonchimeras atlas_repset_nochimera.fasta -chimeras atlas_repset_chimeras.fasta -uchimealns atlas_repset_chimeraalns.fasta -threads 24

usearch v7.0.1001_i86linux64, 264Gb RAM, 48 cores

(C) Copyright 2013 Robert C. Edgar, all rights reserved.

http://drive5.com

Licensed to: robesonms@ornl.gov

00:00 2.6Mb Reading atlas_repset.fasta, 2.3Mb

00:00 4.9Mb 10301 (10.3k) seqs, min 200, avg 200, max 200nt

00:00 5.1Mb Reading sh_refs_qiime_ver7_97_31.01.2016.fasta, 10Mb

00:00  15Mb 17582 (17.6k) seqs, min 141, avg 550, max 2171nt

00:01  16Mb  100.0% Masking

00:01  16Mb  100.0% Word stats

00:01  53Mb  100.0% Building slots

00:03  53Mb  100.0% Build index

00:07 1.2Gb  100.0% Search 137/10301 chimeras found (1.3%)

00:07 1.2Gb  100.0% Writing alignments

00:07 1.2Gb  100.0% Writing 137 chimeras

00:07 1.2Gb  100.0% Writing 10164 non-chimeras

###replace fasta labels with an OTU label###

ava@orion:/data2/ava/fungal_analysis_ATLAS$ python /data2/msr/software/usearch7.0.1001_i86linux64/drive5_py/fasta_number.py atlas_repset_nochimera.fasta > atlas_fungi_repset_nochimeras_OTUs.fasta

###search for sequences prior to dereplication and build an otu map with similarity cutoff at 97% (id)###

ava@orion:/data2/ava/fungal_analysis_ATLAS$ usearch7 -usearch_global trimmed_filtered.fasta -db atlas_fungi_repset_nochimeras_OTUs.fasta -strand plus -id 0.97 -uc all_fungi_otu_map.uc -threads 24

usearch v7.0.1001_i86linux64, 264Gb RAM, 48 cores

(C) Copyright 2013 Robert C. Edgar, all rights reserved.

http://drive5.com

Licensed to: robesonms@ornl.gov

00:00  19Mb Reading atlas_fungi_repset_nochimeras_OTUs.fasta, 2.1Mb

00:00  21Mb 10164 (10.2k) seqs, min 200, avg 200, max 200nt

00:00  22Mb  100.0% Masking

00:00  22Mb  100.0% Word stats

00:00  31Mb  100.0% Building slots

00:00  31Mb  100.0% Build index

02:58 1.1Gb  100.0% Searching, 99.7% matched

###convert to tab delimited###

ava@orion:/data2/ava/fungal_analysis_ATLAS$ python /data2/msr/software/usearch7.0.1001_i86linux64/drive5_py/uc2otutab_mod.py all_fungi_otu_map.uc > atlas_ITS2_OTU_table.txt

###copy to local machine, classify sequences using blast since server doesn’t have correct db for this###

localadmin:~ localadmin$ scp ava@orion:/data2/ava/fungal_analysis_ATLAS/atlas_ITS2_OTU_table.txt /Users/localadmin/Desktop

ava@orion's password:

atlas_ITS2_OTU_table.txt                      100% 7050KB   6.9MB/s   00:01

localadmin:~ localadmin$ biom convert -i /Users/localadmin/Desktop/atlas_ITS2_OTU_table.txt -o /Users/localadmin/Desktop/atlas_fungal_OTU_table.biom --table-type="OTU table" --to-json

localadmin:~ localadmin$ scp ava@orion:/data2/ava/fungal_analysis_ATLAS/atlas_fungi_repset_nochimeras_OTUs.fasta /Users/localadmin/Desktop

ava@orion's password:

atlas_fungi_repset_nochimeras_OTUs.fasta      100% 2074KB   2.0MB/s   00:00

MacQIIME localadmin:~ $ parallel_assign_taxonomy_blast.py -i /Users/localadmin/Desktop/atlas_fungi_repset_nochimeras_OTUs.fasta -o /Users/localadmin/Desktop/Blast_Fungi_ATLAS_Aug2016 -r /Users/localadmin/Desktop/sh_qiime_release_31.01.2016/sh_refs_qiime_ver7_97_31.01.2016.fasta -t /Users/localadmin/Desktop/sh_qiime_release_31.01.2016/sh_taxonomy_qiime_ver7_97_31.01.2016.txt -O 10
